# RNA-Seq Based Identification of Candidate Parasitism Genes of Cereal Cyst Nematode (*Heterodera avenae*) during Incompatible Infection to *Aegilops variabilis*

**DOI:** 10.1371/journal.pone.0141095

**Published:** 2015-10-30

**Authors:** Minghui Zheng, Hai Long, Yun Zhao, Lin Li, Delin Xu, Haili Zhang, Feng Liu, Guangbing Deng, Zhifen Pan, Maoqun Yu

**Affiliations:** 1 Chengdu Institute of Biology, Chinese Academy of Sciences, Chengdu, China; 2 University of the Chinese Academy of Sciences, Beijing, China; 3 College of Life Sciences, Sichuan University, Chengdu, China; 4 Zunyi Medical University, Zunyi, China; 5 Plant Protection College, Shandong Agriculture University, Tai’an, China; East Carolina University, UNITED STATES

## Abstract

One of the reasons for the progressive yield decline observed in cereals production is the rapid build-up of populations of the cereal cyst nematode (CCN, *Heterodera avenae*). These nematodes secrete so-call effectors into their host plant to suppress the plant defense responses, alter plant signaling pathways and then induce the formation of syncytium after infection. However, little is known about its molecular mechanism and parasitism during incompatible infection. To gain insight into its repertoire of parasitism genes, we investigated the transcriptome of the early parasitic second-stage (30 hours, 3 days and 9 days post infection) juveniles of the CCN as well as the CCN infected tissue of the host *Aegilops variabilis* by *Illumina* sequencing. Among all assembled unigenes, 681 putative genes of parasitic nematode were found, in which 56 putative effectors were identified, including novel pioneer genes and genes corresponding to previously reported effectors. All the 681 CCN unigenes were mapped to 229 GO terms and 200 KEGG pathways, including growth, development and several stimulus-related signaling pathways. Sixteen clusters were involved in the CCN unigene expression atlas at the early stages during infection process, and three of which were significantly gene-enriched. Besides, the protein-protein interaction network analysis revealed 35 node unigenes which may play an important role in the plant-CCN interaction. Moreover, in a comparison of differentially expressed genes between the pre-parasitic juveniles and the early parasitic juveniles, we found that hydrolase activity was up-regulated in pre J2s whereas binding activity was upregulated in infective J2s. RT-qPCR analysis on some selected genes showed detectable expression, indicating possible secretion of the proteins and putative role in infection. This study provided better insights into the incompatible interaction between *H*. *avenae* and the host plant *Ae*. *varabilis*. Moreover, RNAi targets with potential lethality were screened out and primarily validated, which provide candidates for engineering-based control of cereal cyst nematode in crops breeding.

## Introduction

Plant parasitic nematodes, consisting of over 4,100 species worldwide, are serious threat to global food security [1]. The complex interaction of plant-parasitic nematodes and their host plants has been studied for several decades. The first “parasitism gene”, encoding β-1,4-endoglucanase, was discovered in 1998, which is capable of degrading cellulose in the plant cell wall and necessary for successful plant infection [2]. Since then, the genes involved in nematode parasitism of plant for several sedentary endoparasitic nematodes, such as *Heterodera glycines*, *Globodera rostochiensis* and *Meloidogyne incognita*, were identified and have been extensively studied [3–5]. These genes are referred as ‘effectors’, in line with other plant pathology discipline. Plant parasitic nematodes use a stylet and secreted effectors to modify host cells and ingest nutrients to support their growth and development. The molecular function of nematode effectors is currently the subject of intense investigation. The identification of putative effectors is a challenging task. As effectors are presumed to be secreted from the nematode into the plant tissue, an effective strategy to discover effectors is to identify nematode secretions directly by proteomics approach. However, this method has drawbacks such as low throughput, high cost and low sensitivity. A more common approach is to investigate the transcriptome of infective or parasitic nematode stages. Bioinformatics tools can subsequently predict putatively secreted proteins, which can be considered as good candidate effectors.

The cereal cyst nematode (CCN) is one of the most severely destructive nematode pests on many important agricultural crops, responsible for an estimated US$125 billion loss in crop yield worldwide annually [4,6]. In China, CCN was found in 16 provinces [7–10]. *H*. *avenae*, one of three dominant species of CCNs, negatively impacted wheat (*Tritium aestivum* L.) production in an area of 40 Mha resulting in significant yield reduction between 15% to 80% annually [11]. However, little is known about the molecular mechanism of *H*. *avenae* parasitism in cereal crops, and far less information about parasitism genes and pathways related to plant-CCN incompatible interaction is available. Comparison of the transcriptome and parasitome among different types of plant-parasitic nematodes is an efficient method to study the parasitic mechanism of plant nematodes [12]. In addition, genomic sequences of some important plant parasitic nematodes were generated in recent years [13–17]. These genetic and genomic resources will provide powerful tools to investigate the parasitism genes of CCN.

Breeding resistant cultivars is the most effective and environmentally friendly way to pest management of *H*. *avenae*. In wheat (*T*. *aestivum*), resistance against the pathotypes of *H*. *avenae* is predominantly provided by the cereal cyst nematode resistant genes (*Cre*). Up till now, 9 *Cre* genes have been found, in which 6 (*Cre*2 to *Cre*7) were derived from *Aegilops* spp [18], the others were derived from *T*. *aestivum* (*Cre*1 and *Cre*8) and *Secale cereale* (*Cre*R) [19,20]. *Ae*. *variabilis* has been documented as a valuable genetic resource for wheat breeding with resistances to both cereal cyst nematodes (CCN, *H*. *avenae*) and root knot nematodes (RKN, *Meloidogyne naasi*) [21,22].

Previous study generated a root transcriptome of *Ae*. *variabilis* during CCN infection [23], which is a promising candidate gene pool aiming at CCN-related genes mining. In this study, we excavated 681 candidate CCN genes from the transcriptome data, which were highly homologous to those from nematode species. We described the gene expression atlas of the early parasitic J2 stage in detail, which the selected three time point were critical during CCN infection[23–27], examined the gene content of *H*. *avenae* in the context of other published plant parasitic nematode genomes or transcriptomes. Then the obtained data here was used to identify the putative effectors or secreted molecules manipulating the host for benefits of nematodes, which provides important in-depth insights into the genes involved particularly in root invasion and establishment of the feeding site, and suggests new anti-parasitic strategies.

## Material and Methods

### Plant material and CCN infection

Before CCN inoculation, grains of *Ae*. *variabilis* were surface-sterilized in a solution containing 3% (v/v) hypochlorite and 0.01% (v/v) Tween 20 for 5 min and rinsed with sterile water for three times. The seeds were germinated in Petri dishes (5cm diameter) on wet paper at 20°C under a 16-h light/8-h dark photoperiod. Ten days later, seedlings were inoculated with newly hatched second-stage juveniles (J2) (1000 juveniles per plant [28,29]. After thirty hours, the roots were thoroughly washed three times with sterile water (each 10 min) to remove the CCNs adhering to roots (S1 Fig). This is to prevent further CCN penetration and ensured synchronized development of syncytia [24,30]. Then all plants were transferred to plastic dishes and grown at 16–22°C under a 16-h light/8-h dark photoperiod. The *Ae*. *variabilis* Accession No. 1 is conserved by our lab and the cysts were collected from the wheat fields of Shandong Agriculture University, Tai’an China (35°53′ N, 116°28′E).

### RNA isolation and RNA-seq sequencing

After the confirmation of successful CCN inoculation and the removing of CCN adhering [23], roots of CCN-infected were sampled for RNA extraction at three time points, i.e. 30 hours post CCN inoculation (hpi), 3 days post inoculation (dpi) and 9 days post inoculation dpi. These three time points are critical for plant resistance during the nematode invasion process. It successfully penetrated roots of *Ae*. *varabilis* at 30-h, then migrated to the stelar regions of the root where the feeding sites (syncytia) had been initiated at 3-d, and lateral root initials were formed by 9-d in the infection process (S1 Fig) [23–27]. Each sample is consisted of 15 individual plants. Total RNA was extracted with a Biomiga RNA kit (Biomiga, San Diego, CA, USA).

The cDNA library construction and sequencings on *Illumina* HiSeq^TM^ 2000 platform [31] were performed at Beijing Genomics Institute (BGI)-Shenzhen, Shenzhen, China, following the manufacturer’s standard protocol. Each library had an insert size of 200 bp, and 50 bp sequences by single-end sequencing were generated as raw data.

### Reads annotation and DEG identification

The sequencing results were annotated based on unigene sequences screening from the root transcriptome database of *Ae*. *variabilis* [23]. After filtering off adaptor-only reads and low-quality reads (reads containing 5 or more ambiguous bases) of the raw data, the remaining reads were mapped on the sequences through the SOAP alignment program [32].

The unigenes expression level was measured as numbers of clean reads mapped on the reference sequence. The number of annotated clean reads for each unigene was calculated and then normalized to RPKM (Reads per Kb per Million reads), which was performed with ERANGE3.1 software and adjusted by a normalized factor [33,34].

To identify differentially expressed unigenes (DEGs), pairwise comparisons were performed between the samples of three stages. False discovery rate (FDR) was applied to judge the significance of the expression difference [35,36]. In this paper, unigenes with at least two-fold differential expression and FDR<0.001 were categorized as statistically significant DEGs.

### Nematode-like unigenes blast search

The DEG unigenes which were only expressed under infection and longer than 200bp (S2 Fig) were Blast (BLASTX) locally against the Swiss-prot and trEMBL protein database (March 2013). In all BLAST searches mentioned above, a bit score of 50 was used as a cut-off to identify significant hits. All publicly available nematode ESTs were downloaded and classified according to the lifestyle of the nematode, resulting in three EST databases of free-living nematodes (FLN), animal-parasitic nematodes (APN) and plant-parasitic nematodes (PPN) (S3 Fig). The data included all sequences available from NCBI’s dbEST, as well as unigenes from recent *Illumina* projects on *H*. *avenae* (S1 Table). A TBLASTX of the *H*. *avenae* unigenes was performed against each of these EST databases to identify parasite-specific sequences. The *H*. *avenae* nucleotide sequences were also searched with BLASTN and BLASTX against the genome contigs data and the predicted proteins, respectively, both downloaded from the genome project websites for *M*. *incognita* and *M*. *hapla* (http://www.inra.fr/meloidogyne_incognita, http://www.hapla.org).

### Effector prediction

Annotation was performed based on the top hit from the BLASTX search result against the Swiss-prot and trEMBL database. Top hits from Swiss-prot sequences with the terms ‘unknown’, ‘putative’, ‘uncharacterized’, ‘hypothetical’, ‘similar’, ‘predicted’ and ‘probable’ in their description were not considered. For the sequences with successful annotation, the protein identifiers of the BLASTX top hits were used to retrieve GO terms using QuickGO (http://www.ebi.ac.uk/QuickGO/GAnnotation). Candidate *H*. *avenae* transcripts were studied for the presence of parasitic effectors identified in other *Heterodera* spp and reported in the published literature. Sequences from known annotated secreted proteins from different plant-parasitic nematodes were downloaded from GenBank (for accession numbers, see S2 Table), as well as the pioneer effector sequences identified in *H*. *avenae* [37]. Peptides potentially secreted were identified using SignalP [38] and those with trans-membrane motifs were removed using TMHMM [39]. SecretomeP 1.0 was used to identify non-classical secretion with default parameters [40]. In all BLAST searches mentioned below, a bit score cut-off of 50 was used.

### Bioinformatics analysis of RNA-seq data

A comprehensive bioinformatics analysis approach was used to enrich the dataset for genes that were most likely to be putative CCN parasitism genes, including: Gene ontology (GO) analysis, KEGG, Biological Analysis Network (BAN), clustering analysis and Protein-Protein interactive analysis.

Go and KEGG analysis GO Annotation and KEGG analysis BLAST2GO was used to map and annotate Gene Ontology(GO) terms [41], with default parameters, except for an E-value cut-off of 1e-6, maximum BLAST hits of 30. KEGG mapping was used to determine the metabolic pathways of metabolism, genetic information processing, environmental information processing, cellular processes, organismal systems and etc. Sequences were submitted to the KEGG Automatic Annotation Server (KAAS) to enrich the pathway annotation.

#### Biological Analysis Network (BAN)

Significant regulated GO categories and KEGG pathways was statistically identified by GO Elite tool with a stringent cut off (FDR<0.05). Unigenes with statistically significant homologies to GO terms and KEGG pathway genes identified by GO Elite tool [42] were used as input for Biological Analysis Network (BAN). Cytoscape v3.2.1[43] was used to cluster the genes and processes using edge weighted force directed (Bio-layout). A network analyzer plugin was used to identify enriched biological categories and regulated genes. Enriched nodes and edges were network modeled using a hierarchical layout algorithm.

#### Cluster analysis

We subjected 649 nematode genes (selected RKPM at all three stage is not zero) to cluster analysis using the Short Time-series Expression Miner (STEM) version 1.3.6 (http://www.cs.cmu.edu/jernst/stem/)[44]. 16 model temporal expression patterns were used to identify the expression patterns of significant differential genes. Each cluster contained certain number of genes that have similar expression patterns after infection. The gene clusters were ranked by the p-value significance of the observed number of genes that fit a profile beyond the expected number.

#### Establishment of PPI network

The protein-protein interaction network of candidate nematode related genes was constructed based on STRING (http://string-db.org/), by using 12 reported parasitism genes as seed genes, which reduced the complexity of the network and confirmed the nod gene. We constructed a network of 118 candidate genes, and listed 35 node genes with the high confidence (≥0.700) based on WormBase and PubMed database.

### Target genes for RNAi

Several subsequent BLAST searches were performed to identify suitable CCN target genes for RNAi. In all BLAST searches, a bit score cut-off of 50 was used, unless stated otherwise. In a first local BLASTX search against all *C*. *elegans* proteins downloaded from WormBase (http://www.wormbase.org, release WS227), the most homologous *C*. *elegans* genes were identified for the *Heterodera avenae* sequences. Next, the Wormbase identifiers of presumably homologous *C*. *elegans* genes were used to retrieve all RNAi phenotypes from the WormMart section of WormBase (release WS220). Genes coupled to lethal phenotypes were subsequently retrieved from the dataset. The sequences of these *C*. *elegans* genes with a lethal RNAi phenotype were downloaded from WormBase and Blast (bit score>40) against the non-redundant protein database including plant sequences only (*Embryophyta*, NCBI txid3193). Genes without hits against the plant sequences were subsequently Blast (bit score>40) against the non-redundant human protein database (NCBI txid9606) and against the non-redundant insect protein database (NCBI txid6960). Only genes with lethal phenotypes and without hits against plant, human and insect sequences were retained as possible targets for RNAi. Three genes were predicted to be interesting targets for RNAi (see Results section). To check whether the silencing of these genes could truly have a lethal effect on the nematode, a soaking experiment with gene-specific siRNAs was set up. The design and construction of the siRNAs were performed according to the Silencer^®^ siRNA construction kit (Invitrogen, Shanghai City, China). Sense (S) and antisense (AS) primers that were used as templates were as follows: Unigene_38116_siRNA_AS, CAACCTGCACCGAATACTTCACTACAAA; Unigene38116_siRNA_S, ACCCTAAATAATGGAGACCTCACTAACG; Unigene102492_siRNA_AS, ACAAGATGACGGAAATGGAAGAAGAGTT; Unigene102492_siRNA_S, CGTTAGTGAGGTCTCCATTATTTAGGGT; Unigene38007_siRNA_AS, AAGAGCCAACAATCTCCGAGTTCTCCCT; Unigene38007_siRNA_S, CACCAAGACCAACTACCGAACCACAAGA; GFP_siRNA_AS, AAACATTCTCGGCCACAAGCTCCTGTCTC; GFP_siRNA_S, AAAGCTTGTGGCCGAGAATGTCCTGTCTC. The collected cysts were hatched at 15°C in an artificial climate incubator. After 2 days of extraction, parasitic J2s were collected and soaked for 24 h with gene-specific siRNAs at a final concentration of 25 ng/μl. As control treatments, nematodes were soaked in water or in 25 ng/μl siRNAs targeting GFP. After soaking, the viability of the worms was evaluated by counting the number of living and dead nematodes in each sample using light microscope. Statistical comparisons between samples were made using the Kruskal-Wallis-test in SPSS software version 20 (Chicago, IL, USA) (P<0.05). This experiment was repeated thrice.

### Real-time quantitative PCR (RT-qPCR)

RT-qPCR was performed to validate the RNA-Seq result. Total RNAs of various samples were re-isolated from the CCN-infected root of 15 plants of *Ae*. *variabilis*. cDNAs of the three samples were synthesized with oligo-dT primers. Six parasitism gene candidates were selected for primers design based on the sequence information in the 3’ UTR [45]. Transcriptional profiles using the iQ^TM^ SYBR^®^ Green Supermix (Bio-Rad) and a Bio-Rad Chromo 4^TM^ CFB-3240 system (Bio-Rad Lab. Inc., Hercules, CA, USA). The PCR mixture contained 200ng cDNA, 10 μl of iQ^TM^ SYBR^®^ Green Supermix (Bio-Rad) and 300 nM each primer in a final volume of 20 μl. The PCR cycling parameters were set at 95°C for 20s, followed by 40 cycles of 95°C for 25 s, 58°C for 25s and 72°C for 20s. After completion of the cycling parameters, dissociation melt curve analysis (65–95°C every 0.5°C for 5s) was conducted to discount the effects of primer dimer formation and contamination. Two reference genes identified in the unigenes were used; an FMRF amide-like neuropeptide 14 and an elongation factor 1α. Primers are listed in S3 Table. All reactions were performed in triplicate and the negative controls included water and mRNA prior to reverse transcription [46,47]. Data were analyzed based on the stable expression level of the reference genes [48].

## Results

### *Illumina* sequencing and unigene mining of *H*. *avenae* J2s during infection

The single end RNA-seq sequencing using *Illumina* technology resulted in over 70,992,271 robust reads with an average length of 50 bp. After cleaning, adaptor trimming, and assembly, 481,672 unigenes longer than 200 bp were harvest (Table 1). The average length of the assembled unigenes is 525 bp, ranging from 200 to 3056 bp. A BLASTX search against the Swiss-prot and trEMBL databases revealed that 846 sequences showed significant similarity to know nematode sequences (Table 1, S4 Table), in which 812 unigenes were also supported by nematode ESTs (S3 Fig) from three smaller datasets of free-living nematodes, animal-parasitic nematodes, and plant-parasitic nematodes. Out of the all 812 candidate unigenes, 681 unigenes showed similarity exclusively to those of the plant parasitic nematode (e-value ≤ 1e-5) (S5 Table).

**Table 1:** Overview of sequencing run and assembly.

	Number	Total bases (Mbp)	Average length(bp)
**Reads**	70,992,271	4,687.31	50
**Contigs**	481,672	92.50	192
**Unigenes**	118,064	59.09	500
**CCNUnigenes ≥200bp**	846	0.42	200

The root-knot nematodes and cyst nematodes, causing the largest economic losses to agriculture, belong to the order *Tylenchida*. Of which, the genus *Meloidogynei* is relatively well studied. Therefore, we compared the 681 unigenes with the genome sequences of *M*. *incognita* and *M*. *hapla*. As a result, 235 and 189 orthologs were found in *M*. *incognita* and *M*. *hapla*, respectively (S6 Table). By comparing to the transcriptome of *H*. *avenae* [14], 525 orthologous were found (S5 and S6 Tables).

### GO and KEGG analysis

The 681 unigenes could be annotated to 229 GO terms, among which 116 (grouped in 83 subcategories), 54 (grouped in 30 subcategories), and 59 (grouped in 30 subcategories) GO terms could be grouped to the biological process category, the molecular function category and the cellular component category consisted, respectively. In our analysis, most of the annotated genes occurred in the cytoplasm, involved in functions of nucleotide binding or transport. In the ‘cellular component’ category, unigenes annotated to “the cytoplasm” term were most abundant, whereas unigenes linked to the “membrane” occurred much less frequently. In the ‘molecular function’ category, the most over-represented terms were “binding”, “nucleotide binding” and “catalytic activity”, whereas “receptor” and “signal transducer activity” were the under-represented terms. With regard to the ‘biological process’ category, the most over-representation of terms are related to larval growth and development, whereas sensory perception and the regulation of transcription and metabolic processes were under-represented (S4 Fig).

**Fig 1:**
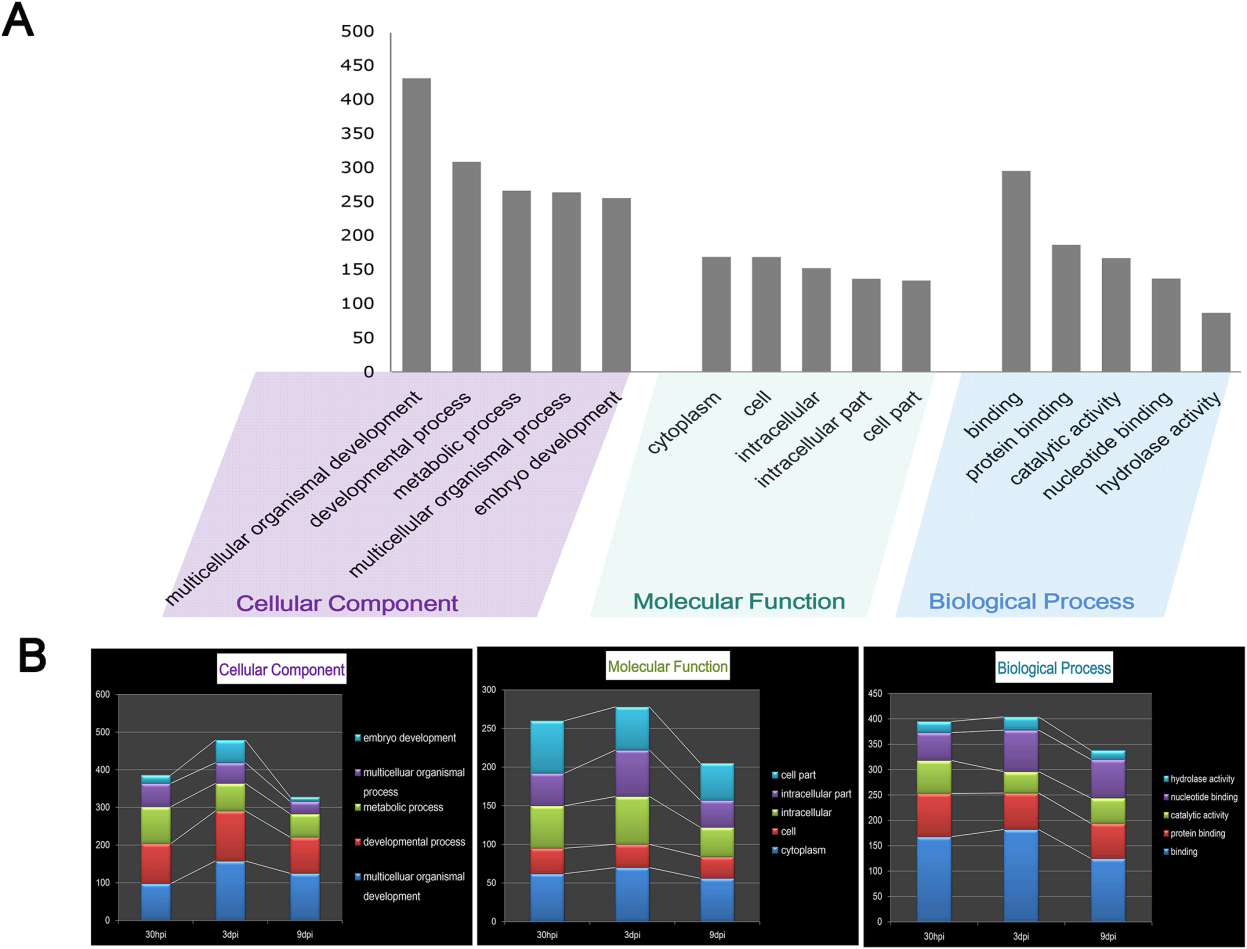
Numbers of unigenes in occurrence of the top five gene ontology (GO) terms and the dynamic changes of these GO terms associated with disease progression. A. Numbers of unigenes of the top five GO terms in the categories ‘cellular component’, ‘molecular function’ and ‘biological process’. B. Dynamic changes of these GO terms associated with disease progression.

The most economically important cyst nematode species are within the *Heterodera* and *Globodera* genera. We then took a reciprocal BLAST strategy using the GO associations data of species in the two genera (*G*. *pallid*, *G*. *rostochiensis*, *H*. *schachtii*, *H*. *glycines*, *H*. *avenae*) available on the Nematode.net and HATdb (Table 2). The top five GO terms in the comparison were showed in Fig 1A, and the dynamic changes of these GO terms in our dataset associated with disease progression were showed in Fig 1B.

**Table 2:** The summary of parallel comparison of the GO terms between the different genus of nematode.

Genus	No. of Contigs or Unigenes	Biological process	Cellular component	Molecular function
***H*.*avenae* (J2s)**	681	499	374	459
***H*. *avenae***	27765	NA	NA	NA
***G*. *pallida***	2973	624	333	849
***G*. *rostochiensis***	2530	651	394	869
***H*.*glycines***	2026	1887	978	2337
***H*.*schachtii***	1600	371	210	457

**Fig 2:**
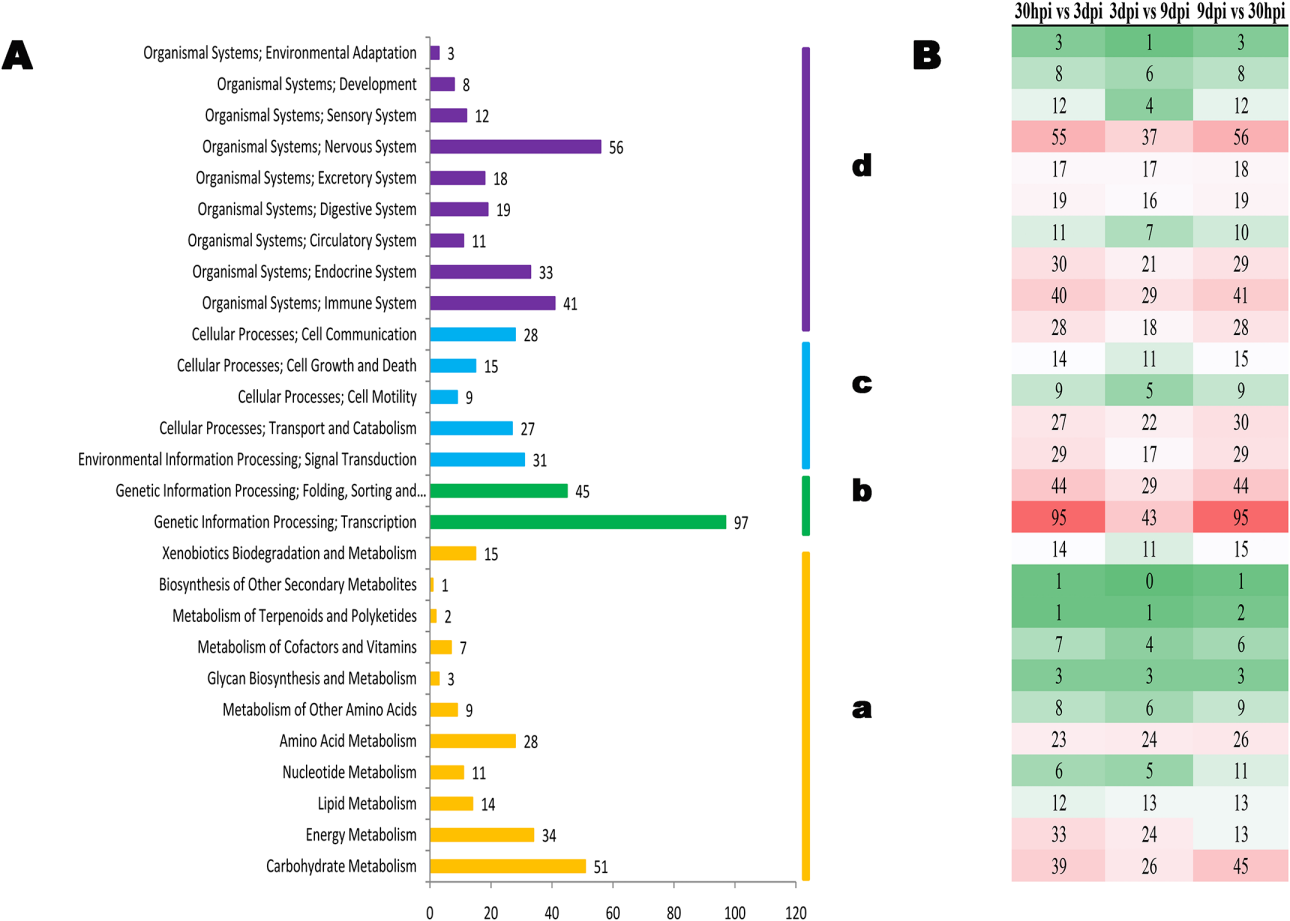
KEGG classification and DEGs including in each category at the three time points. A. KEGG classification a, Primary Metabolism; b, Genetic Information Processing; c, Cellular Process; d. Organismal Systems. B. DEGs including each category among all the three time.

**Fig 3:**
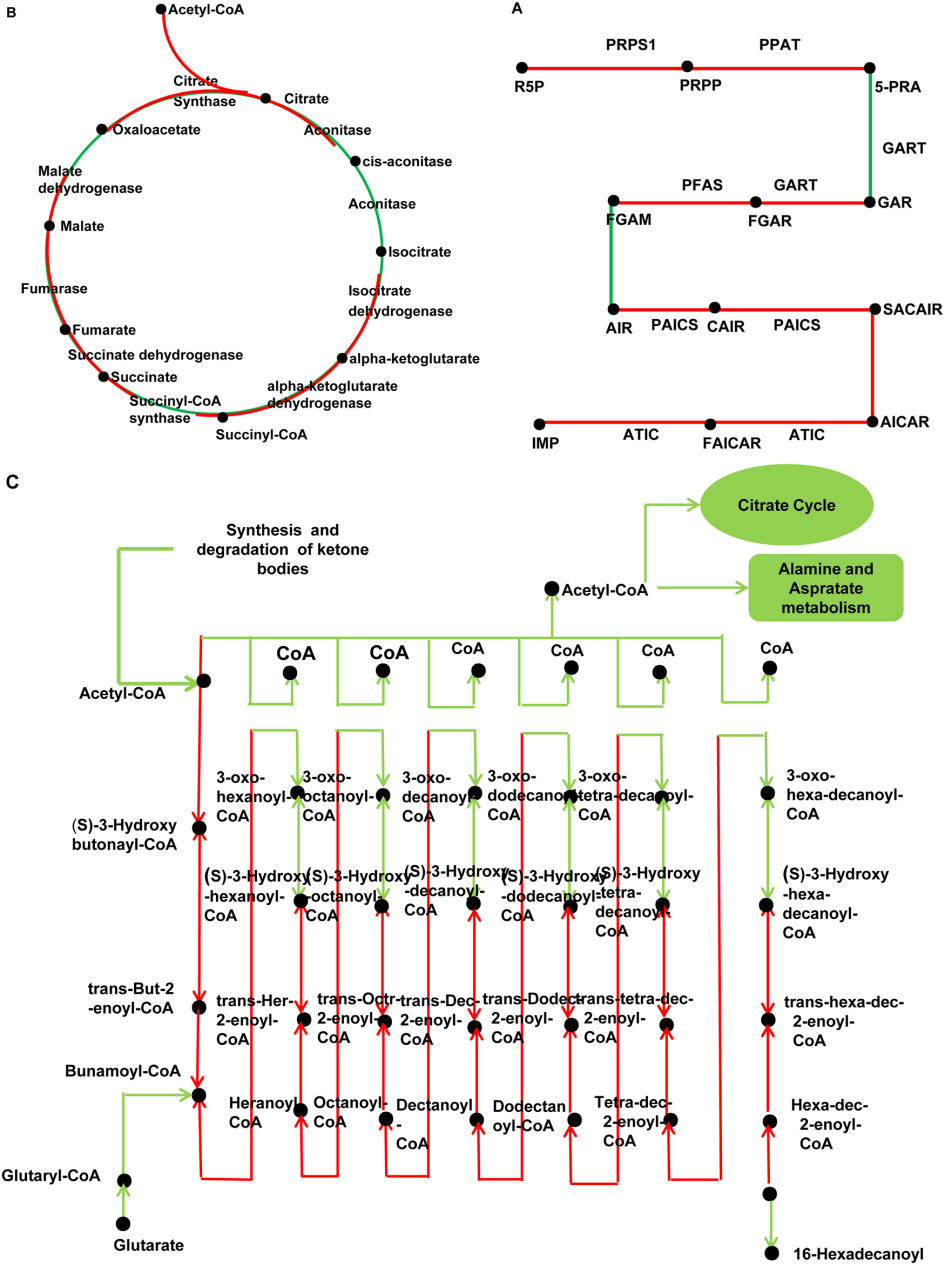
Interactive pathways analysis of genes during the infection of *Heterodera avenae*. The red lines indicate genes can be found in our data. A, Purine Metabolism; B, Citrate cycle; C, Fatty acid metabolism.

As an alternative method of categorizing cluster sequences by biochemical function, sequences were assigned to biological pathways using the Kyoto Encyclopedia of Genes and Genomes (KEGG) database (http://www.genome.ad.jp/kegg). All 681 unigenes could be clustered into 200 pathways (Figs 2, 3 and S7 Table), in which the most over-represented pathways are metabolic pathways (175), carbohydrate metabolism (51), biosynthesis of secondary metabolites (24), oxidative phosphorylation (16), and ubiquitin mediated proteolysis (10). The most significant enriched KEGG pathways were purine metabolism (Fig 3A), citrate cycle and fatty acid metabolism (Fig 3B), which are the basal metabolism of nematodes (Fig 3C). Besides, there were 14 KEGG Orthology (KO) mapped to the lipid biosynthesis pathway.

**Fig 4:**
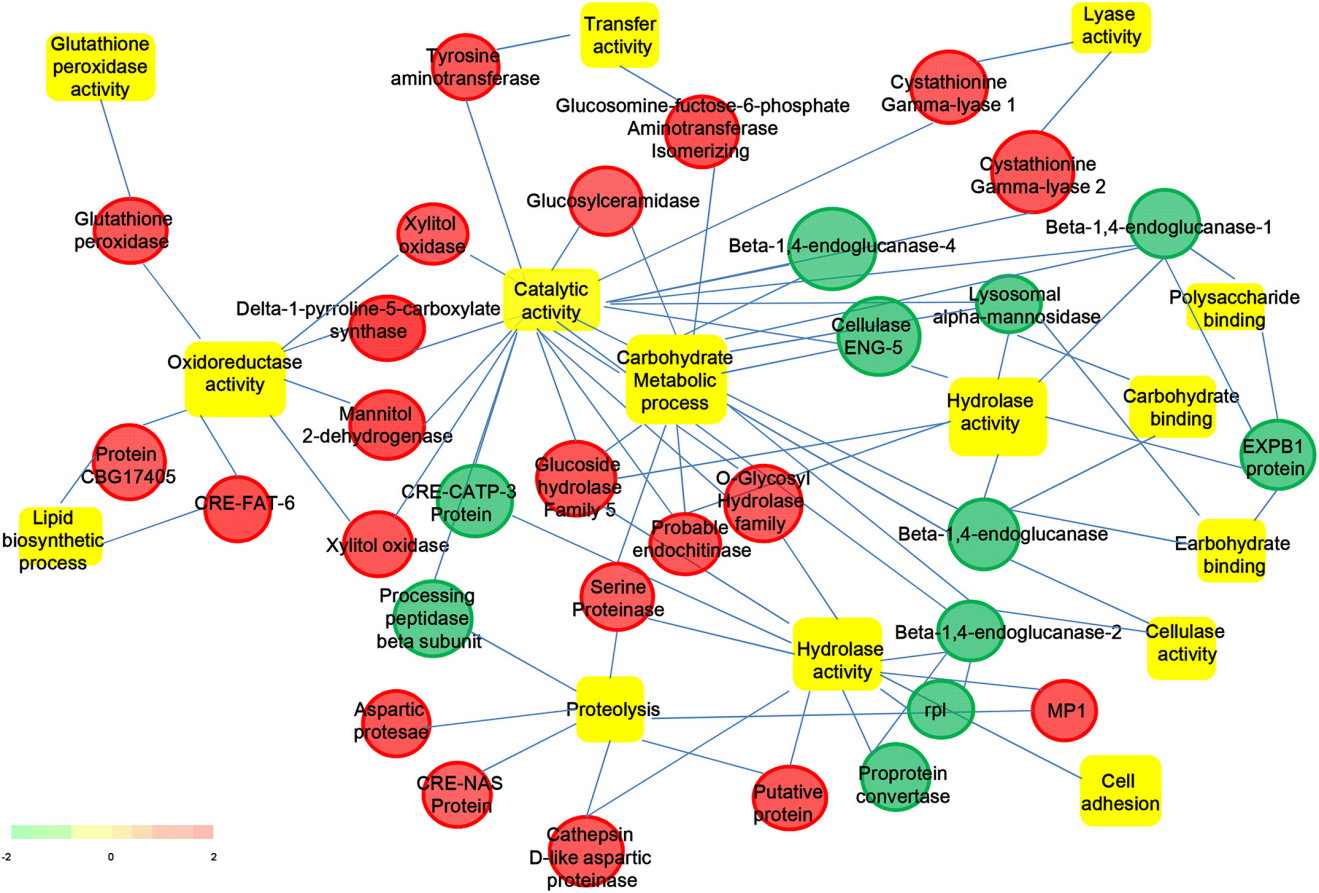
Hierarchical layout of significantly enriched biological processes, pathways and key regulatory genes in *H*. *avenae*.

To highlight the distinct biology (GO terms or KEGG pathways) of cyst nematodes during the infection process, comparison were conducted between the pre-parasitic J2s and the early infective J2s. Regulated unigenes were subjected to GO term enrichment analysis using GO-Elite V1.2.5 (http://www.genmapp.org/go_elite/). Analysis revealed 62 biological categories under GO terms and pathways significantly enriched at the stringent cut off (FDR<0.05) (Table 3). To identify the major functional themes, enriched GO terms or pathways were organized into a functional network with 32 nodes and 12 edges using enrichment map plugin in Cytoscape v3.2.1 (Fig 4). Four functional clusters including a) carbohydrate binding, b) proteolysis, c) motor activity, and d) hydrolase activity, were showed by FDR-based sub-clustering with the enrichment map method based on force directed layout visualization (Fig 4). And visualization of sub-clusters using hierarchical layout suggested that hydrolase activity was up-regulated in pre-parasitic J2s whereas binding activity was up-regulated in early parasitic infective J2s.

**Table 3:** DEG enriched GO categories and pathways between the root-free pre-J2s and infective J2s of *H*. *avenae*.

**Category**	**Term**	**Z-Score**	**p-value**
**KEGG**	ko02010	4.38	0.00
**KEGG**	ko01212	7.03	0.00
**KEGG**	ko00650	3.65	0.02
**Cellular component**	GO:0016021	1.52	0.22
**Biological process**	GO:0006508	1.33	0.16
**Biological process**	GO:0005975	2.88	0.04
**Molecular function**	GO:0004601	3.55	0.03
**Molecular function**	GO:0030246	5.26	0.00
**Biological process**	GO:0006633	1.95	0.11
**Biological process**	GO:0006979	4.33	0.00
**Molecular function**	GO:0004553	6.43	0.00
**Cellular component**	GO:0016021	1.85	0.23

### Effectors prediction

Plant-parasitic nematode effectors, defined as proteins secreted by the nematode into the host and manipulating the host for benefits of nematodes, are usually expressed in the sub-ventral or dorsal pharyngeal gland cells and then secreted into the host via the stylet [49]. Unigenes with predicted signal peptides and lacking trans-membrane domains were identified by TMHMM (TransMembrane prediction using Hidden Markov Models) [50]. Totally, 323 putative effectors were identified, of which 213 showing significant similarity with sequences of the host or microbe were excluded. Among these candidate homologues, 56 unigenes were specifically similar to those of plant-parasitic nematode effectors, and 5 unigenes were unique to *Heterodera* spp (Table 4).

**Table 4:** Important genes identified in *H*. *avenae* based on best BLASTX hits.

Accession No .	Descriptions	Species	e -Value	Identity (%)	Reference
**ACO55952**	beta-1,4-endoglucanase	*Heterodera avenae*	3.00E-93	97	[69]
**ADD82848**	Pectate lyase	*Heterodera avenae*	3.00E-17	81	[70]
**ACV31368**	Expansin	*Heterodera glycines*	1.00E-26	75	[71]
**ACN93668**	*Annexin*	*Heterodera schachtii*	1.00E-130	58	[72]
**CAD38523**	Glutathione peroxidase	*Heterodera glycines*	2.00E-44	65	[73]
**I3VB56**	Calreticulin	*Radopholus similis*	4.00E-61	91	[74]
**ADY48649**	Ubiquitin-conjugating enzyme E2	*Ascaris suum*	4.50E-81	59	[75]

Effectors with homology to the known genes involved in the parasitic interaction were identified, such as carbohydrate-active enzymes (CAZymes), which are important in nematode migration in the plant and feeding from host cells [51]. By using the CAZymes Analysis Toolkit (CAT) [52], We found 23 plant cell degradation-related CAZymes, such as pectate lyase, expansin, and cellulose-binding protein, etc. Moreover, in plant parasitic nematodes, antioxidant enzymes may provide protection against plant defenses [53]. We found 10 unigenes encoding the homologues of secreted proteins involved in detoxification of reactive oxygen species (ROS) (such as *peroxiredoxin*, *glutathione peroxidase*, *glutathione-S-transferase*). Other putative effectors, including fatty acid and retinol-binding protein, annexin, calreticulin, chitinase, transthyretin-like protein, mitogen-activated protein (MAP-1), C-type lectin and 14-3-3 protein, which are important in the infection or development process of the plant parasitic nematodes, were also found (S2 Table).

### Expression cluster analysis

**Fig 5:**
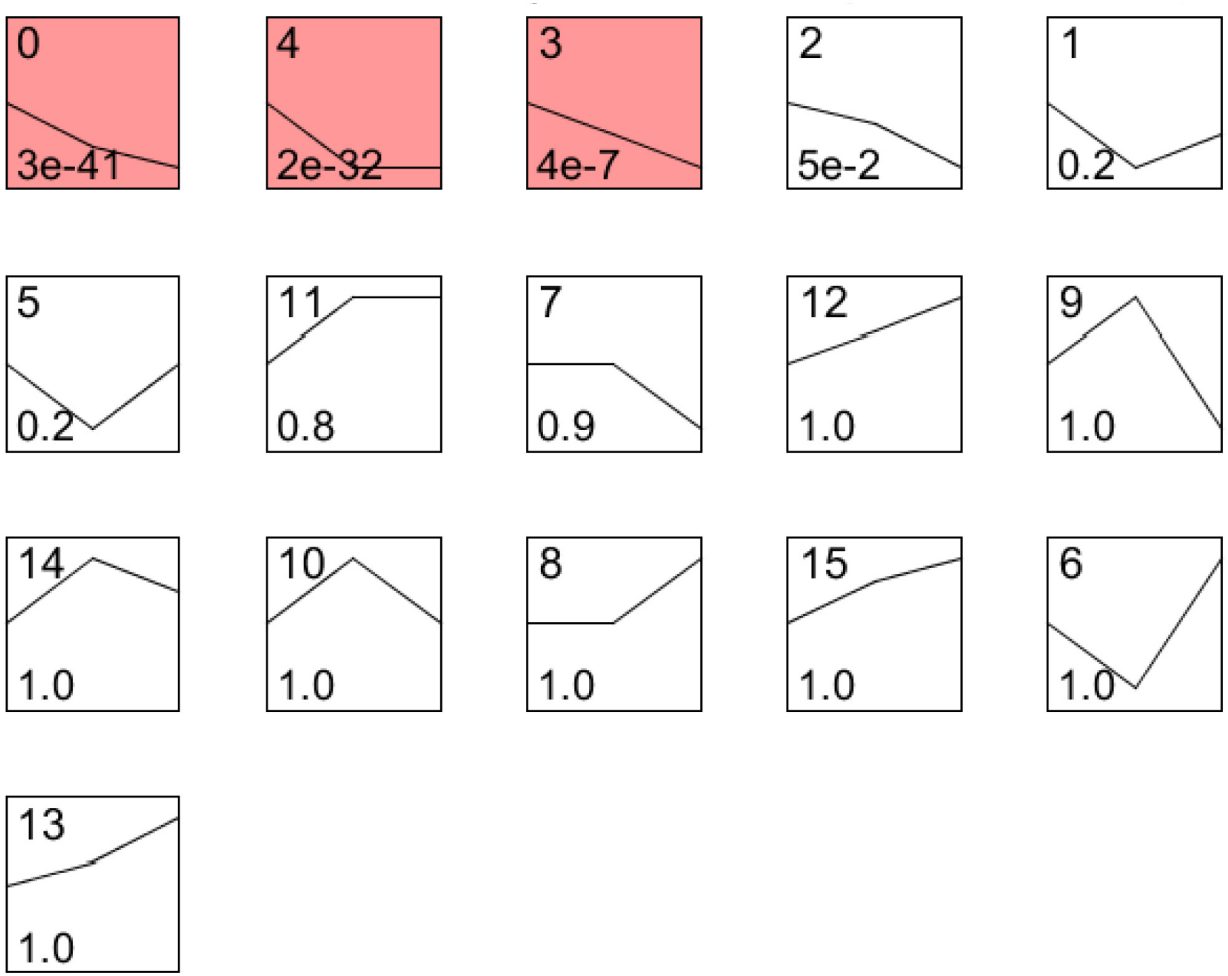
Temporal expression patterns of *H*. *avenae* parasitism gene using STEM. Profiles ordered based on the p-value significance of number of genes assigned versus expected. All 649 *H*. *avenae* parasitism gene were differentially expressed (FDR = 5%) over the three infective stage and were grouped into 16 clusters. The average expression pattern of each cluster is represented by a bold line, Three profiles (Red background p<0.001) with a statistically significant number of genes assigned. The number in the top corner represents the ID of the profile; the curve shows individual gene expression profiles; the number in the left corner represents the Significance.

**Fig 6:**
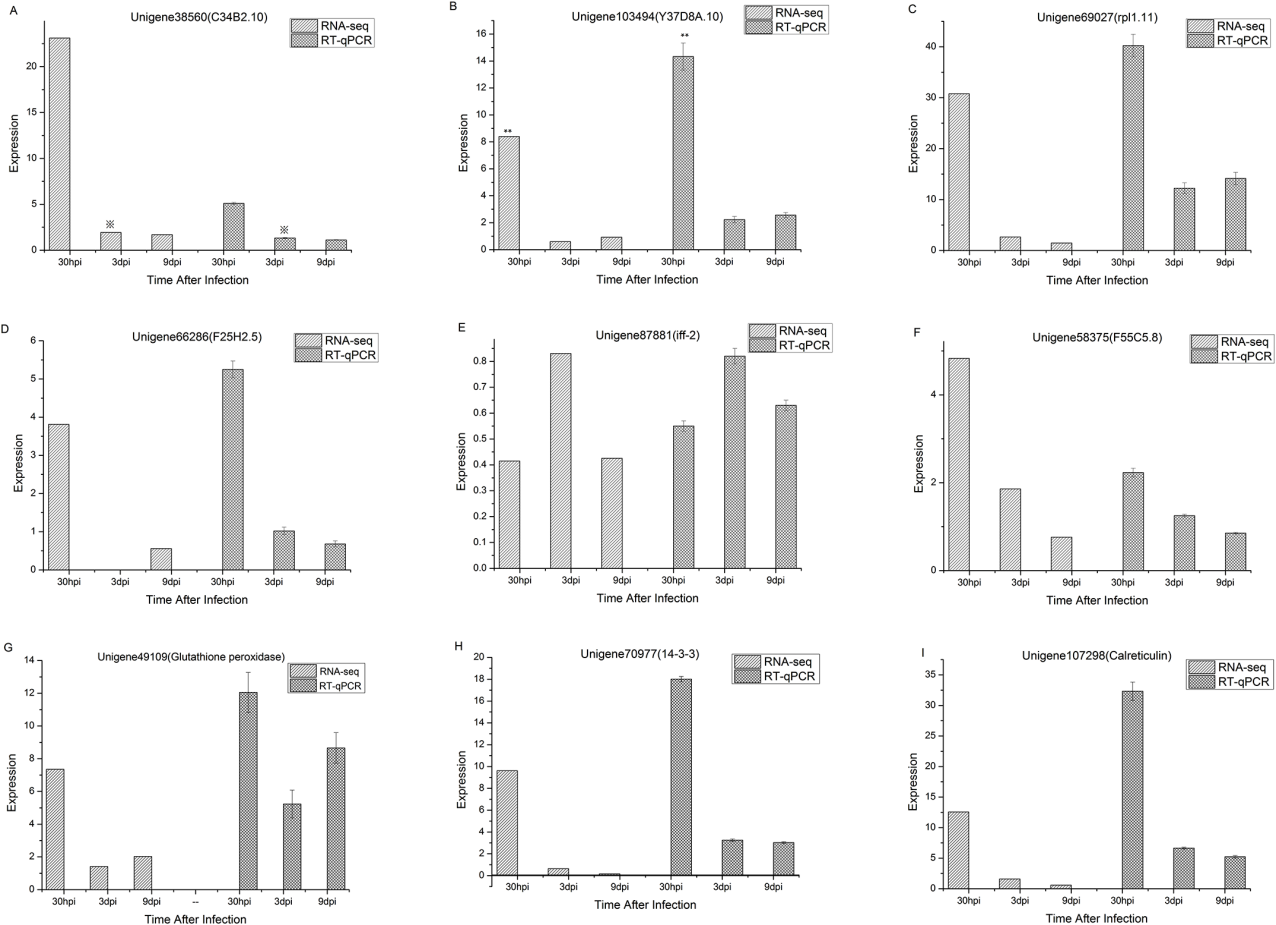
Validation of RNA-seq results by qPCR after infection. A~F show the expression fold changes of six selected nematode parasitism genes: *C34B2*.*10*, *Y37D8A*.*10*, *rpl-11*.*1*, *F25H2*.*5*, *iff-2* and *F55C5*.*8* at 30h, 3 and 9 DAI; G, H, and I show the expression fold changes of three effector genes (error bar represents one s. d.). The relative changes in gene expression were calculated by the 2 –^ΔΔCt^ method, with FMRF amide-like neuropeptide 14 and an elongation factor 1α serving as an internal control gene.

Different parasitism genes are pools deployed at different phases of parasitism. Therefore, study of expression patterns of CCN parasitism genes can provide a promising candidate for identifying effectors [54]. We then examined the temporal expression patterns of the 681 unigenes at early parasitic stages: 30hour, 3days and 9days post infection (hpi, dpi). Genes showing similar expression patterns during CCN infection were clustered. Three significantly enriched clusters containing a total of 271 unigenes were identified (P<0.001) (Fig 5, Table 5, S8 Table). All unigenes in the three clusters are highly expressed at early time points, then were gradually descended and repressed at later time points. Out of the 271 candidate parasitism genes, 36 did not show similarities with known sequences. The quantitive real-time PCR (qRT-PCR) was employed to validate the accuracy of gene expression levels calculated from RNA-Seq data. It showed that the 9 randomly chosen unigenes exhibited consistent expression patterns crossing the three time points as those of RNA-seq (Fig 6).

**Table 5:** The summary of critical functional categories in significant expression patterns.

Function Category	Profile		
	0	4	3
**Developmental process**	√		
**Metabolic process**	√	√	√
**Multicellular organismal process**	√		√
**Response to stimuli**		√	
**Embryo development**		√	√

### Protein-protein interaction (PPI) network of obtained *H*. *avenae* genes

**Fig 7:**
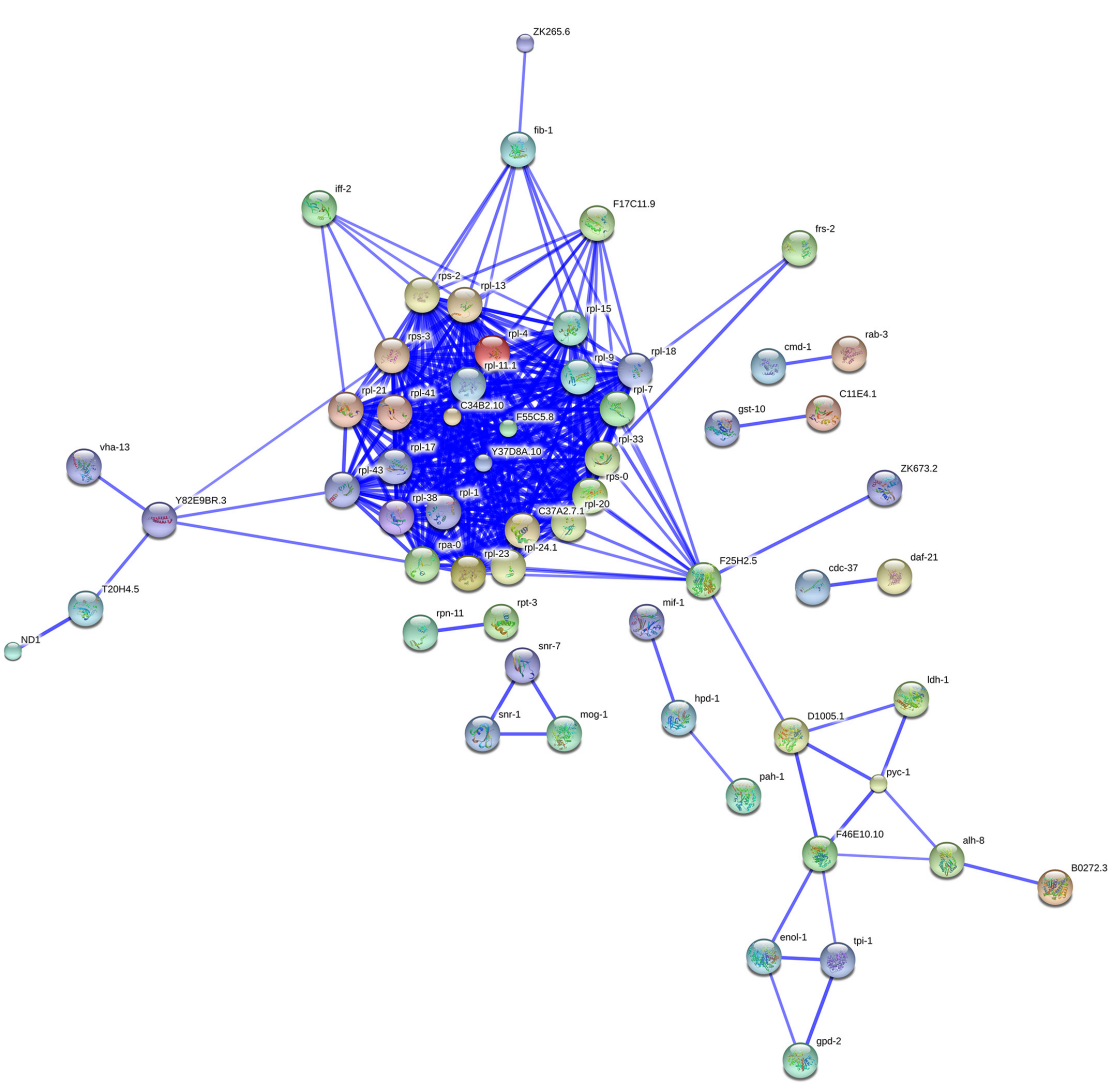
The PPI network constructed based on STRING DATA. The 118 overlapping genes were used as seed genes to construct a PPI subnetwork. Network Nodes represent the overlapping genes. Edges between them represent the protein-protein interaction. All information is based on STRING DATA (High Confidence (0.700)).

To further mine putative pivotal parasitism genes during CCN infection, we performed protein-protein interaction network analysis (PPI) in accordance with following procedures: First, nematode parasitism genes were extracted through WormBase and PubMed database queries using following keywords: plant-parasitic nematode, parasitic stage, parasitism gene and effector. Second, 118 candidate genes were found in our unigene set (S9 Table). Third, a comparison between our experimentally derived parasitism genes and text-mined genes was conducted. The comparison showed that 68 unigenes in our dataset were confirmed to be involved in plant-CCN interaction. These genes are defined as overlapping genes, in which 12 reported parasitism gene were selected as seeds to perform PPI analysis. The protein-protein interaction (PPI) network was constructed based on the STRING Database to depict their complex relationship (Fig 7), in which 35 nodes were found in our PPI network.

### Identification of candidate genes for RNA interference (RNAi)

**Fig 8:**
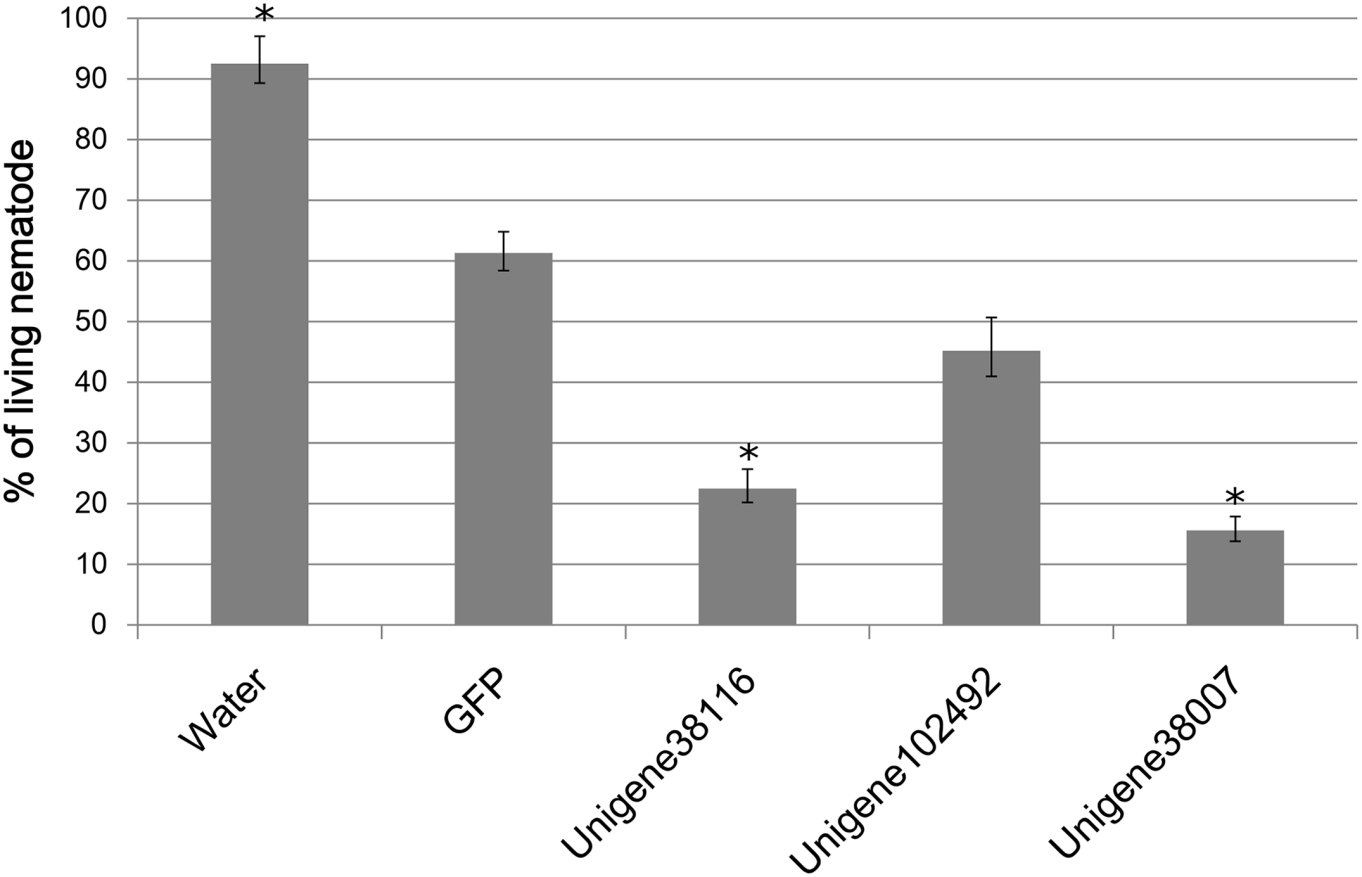
Percentage of living nematodes and microscopic examination (DFC450 Leica) after 24 h of soaking with gene-specific small interfering RNAs (siRNAs) (25ng/μL). Treatments that are significantly different (P<0.05) from the green fluorescent protein (GFP) treatment are indicated by stars. A, H_2_O; B, Unigene38116; C, Ungiene102492; D, Unigene38007.

RNAi is one of the proposed strategies to control plant-parasitic nematodes [55,56]. To identify putative RNAi targets, we searched *C*. *elegans* genome for homologues with the following criteria: (a) homologous to *C*. *elegans* genes having a lethal RNAi phenotype; (b) relatively highly expressed; and (c) don’t showing significant similarity to sequences from plants, insects or humans. As a result, 16 unigenes were screened out, in which three functionally unknown candidates supported by over 20 reads in our RNA-seq dataset were chosen as targets for small interfering RNAs (siRNAs) based lethality evaluation (Table 6). The results showed that except for unigene102492, the CCN treated by siRNAs targeting unigene38116 and unigene38007 showed significantly higher mortalities than those treated by green fluorescence protein (GFP) or water (Fig 8 and S5 Fig).

**Table 6:** CCN Unigenes chosen as RNA interference (RNAi) targets. Because they have a lethal RNAi phenotype in *Caenorhabditis elegans*, but do not show similarity to plant, human and insect sequences. The CCN ungiene, the *C*. *elegans* homologue, number of reads, Its RNAi phenotype and the description of the *C*. *elegans* proteins are shown.

Unigene	Reads	*C*. *elegans* homologue	RNAi phenotype	Description
**Unigene38116**	42	*C18D1*.*3*	Larval lethal	*FLP*-4
**Unigene102492**	21	*F38E11*.*7*	Embryonic lethal	*WRT*-3, isoform a
**Unigene38007**	25	*C06G4*.*2*	Larval lethal	*Calpain* homolog

## Discussions

*H*. *avenae* is an economically important pathogen of cereals, as well as a key model system for understanding the biology of cyst nematodes, one of the most important groups of plant pathogens worldwide [57]. Excavation of the molecular mechanism underlying interactions of *H*. *avenae* and host plant is essential for better understanding of the biological essence of cyst nematode parasitism, which will facilitate the CCN prevention by traditional breeding program or bioengineering strategies. To achieve this purpose, the primary step is to identify pivotal genes involved in diverse processes related to parasitism. Recent genomic and transcriptional analyses have extended parasitism identification to phytonematode species including some migratory plant parasites [58–60]. In this study, by using RNA-seq technology of *Illumina*, we produced over 481,672 unigenes derived from the infected *Ae*. *varabilis* root and excavated 681 exclusively unigenes of *H*. *avenae*. To our knowledge, this is the first study to identify the parasitism-associated genes of *H*. *avenae* in the early parasitic J2 stages (30 hour, 3 days, and 9 days post infection) during incompatible infection. And sedentary endo-parasitic nematodes like the *H*.*avenae* do not secrete the vast number of parasitism proteins until they have invaded their host plants. Comparative analyses of transcriptome data derived from the pre-parasitic J2s and parasitic J2s, and sequences of other nematode species revealed that 34.5% (235), 27.7% (189), and 77.1% (525) of the obtained *H*. *avena*e unigenes are homologous to those of the well-studied *M*. *incognita* and *M*. *hapla*, and pre-parasitic J2s, respectively. However, 156 parasitism genes obtained in this study were not found in the *H*. *avenae* transcriptome published recently [14]. This may due to the incomplete transcriptome data set or some of these genes are specifically expressed during interaction with host.

Assignment of GO terms and KEGG pathway categorised all 681 transcripts to putative functions. By checking the over-represented and under-represented GO terms, we found that a high number of sequences are involved in nucleotide binding, as well as numerous sequences with catalytic, phosphatase, hydrolase and ATPase activity. Moreover, owing to at the early parasitic J2 stage, terms related to larval development and growth and development were also over-represented. Function annotations indicated that these unigenes may play a role in the pathogenesis during the process of infection or development of feeding site. While enriched KEGG pathways for clusters provided a broad view of the potential functions of molecules with stage-dependent expression patterns (379), such as carbohydrate metabolism (51), oxidative phosphorylation (36), ubiquitin mediated proteolysis (16) and the lipid biosynthesis pathway (14). Based on the function explanations, we assumed that some pathways are probably important in parasitism-related processes. For example, lipid biosynthesis are the primary source of energy in eggs of plant-parasitic nematodes (PPNs), such as *M*. *hapla* and *M*. *incognita*, who secrete a specific class of fatty-acid and retinol-binding (FAR) proteins that may interfere with lipid-based defenses by inhibiting the production of jasmonic acid in the host [61,62]. Besides, glutathione and peroxiredoxin are key steps to scavenge the cytotoxic hydrogen peroxide released by host plant during infection process [63]. And ubiquitination [64,65] has been implicated in regulation of many processes in plants, such as innate immunity, cell death, cell cycle regulation, hormone signaling and circadian rhythms. Moreover, when comparing the temporal expression patterns of the root-free pre-parasitic J2s and the early parasitic J2s identified here, it became evident up-regulation that hydrolase activity was in pre-parasitic J2s whereas binding activity was up-regulated in early parasitic infective J2.

To be a successful plant pathogen, *H*. *avenae* must locate a host plant root, enter it using its mouth stylet, and migrate from cell-to-cell before inducing the feeding site syncytium [14]. As found for other plant endo-parasitic nematodes, a range of effectors have been identified which are possibly required to invade into root, modify plant cell walls during migration, feeding or syncytium formation. Then by analyzed the root transcriptome during incompatible infection, we identified 56 putative effectors, which may play important roles in parasitism, such as plant cell wall-modifying proteins [37]. Series of cell wall-modifying enzymes, such as *xylanase* and *expansins*, were also identified in our dataset. These enzymes were thought to be involved in parasitic J2s migration to the final feeding site, and had been found in many other plant endo-parasitic nematodes, such as *M*. *incognita*.

Genes important for the interaction between the nematode and its host are likely to be secreted into host tissues or cells. To predict putative secreted proteins encoded by *H*. *avenae*, we identified 122 unigenes with signal peptides but don’t contain trans-membrane domain. Some of such unigenes have no matches in any free living nematodes, which therefore might be related to the interaction of *H*. *avenae* with its host. These genes may be good candidates of novel new effectors and need be further validated. Since parasitic nematodes have to suppress different host defense responses for their survival, they may achieve these using different mechanisms. The C-type lectin found in *H*. *avenae* is an interesting gene that might be involved in overcoming the host defenses as reported in animal parasitic nematodes [66]. Also we found unigenes for the enzyme *glutathione reductase* in our unigene set that may provide protection to *H*. *avenae* against ROS, which may have the same function as ones in animal parasitic nematodes [67].

Clustering of gene expression profiles may reflect changes in the transcript profiles in *H*. *avenae* across early parasitic stage. Nearly 40% (271) of the identified unigenes were grouped into the three distinct expression clusters comprising of representing early up-regulated patterns. The cluster profile 0 comprises 105 differentially expressed unigenes. GO terms significantly enriched in this cluster were all related to stimuli response and development process. Similarly, 47 unigenes of the cluster profile 4 were significantly enriched for GO terms related to cellular processes (cell proliferation, cell cycle, cell adhesion) and signaling processes, reflecting the fact that these life stages are actively feeding and undergoing feeding site’s developing.

Alternatively, clusters of the secretome up-regulated in the later stage (9days post infection) might be of interest because the genes in these groups are down-regulated in early parasitic J2 stages (30hour and 3days post infection). The expression profiles of these genes should be a clear down-regulation after the parasitic J2 stage, which is the beginning of the sedentary phase of the nematode and initiation of the feeding site development [68]. However, the down-regulation expression profiles in our data during early parasitic J2 stage may indicate that the CCN’s infection was suppressed by host plant’s resistance response.

By PPI network analysis (Fig 5), putative key parasitism genes at the node positions were identified. Some of these genes are likely to participate in the development process, such as carbohydrate metabolism, which is upregulated in the transition to the hatched J2s, and then may play an important role in the following later parasitic J2 stage. Some node genes encode potential effectors, such as fatty acid retinoid binding protein RANBP-like protein (unigene110116_1), pectate lyase (unigene89846_1), esophageal gland cell secretory protein (unigene92768_1) and *Annexin* (unigene69180_1), function as enzymes modifying plant-cell wall.

Moreover, some node parasitism genes in the significantly enriched expression profile may be of importance in infection or metabolism process during the early parasitic J2 stage, such as serine protease, β-1-4-endoglucanase, rpl-11.1, rpl-24.1, iff-2, and F55C5.8. And some randomly selected ones among were checked by qRT-PCR and the relative high expression supported the suggestion that in the infective J2s some of these gene products may be required for host invasion. Since *H*. *avenae* may survive in the soil for several years, it is of interest to understand its mechanism of survival and development. In our PPI network, most of the unigenes linked to the basal metabolism and develop process. While their expressions were obviously suppressed at the later time point (3 days), which might be due to the triggered defense response of *Ae*. *varabilis* [46]. Taken together, the protein-protein interaction networks provide basic information for CCN’s parasitism mechanism during early parasitic J2s, which could act as an initial step for better deciphering the molecular mechanisms of plant-CCN interaction together with our transcriptome results.

The genes essential for the nematode’s survival are ideal targets for parasite control by using reversed genetics strategies, such as RNAi. Sixteen candidate RNAi targets were predicted in this study. Soaking experiments for among three genes showed that gene-specific siRNAs indeed had a lethal effect on the nematodes, with up to 40% less survival compared with controls. These results confirmed the potential utilization of these genes in engineering improvement of CCN resistance in crops.

## Conclusions

In conclusion, we have undertaken the first transcriptome analysis of the early parasitic J2 stages of *H*. *avenae* during incompatible infection, and identified some genes that may be important in plant parasitism. Additionally, a comparative analysis of gene expression between the pre-parasitic J2s and the early parasitic J2s provides additional information on some pivotal genes that are likely to be involved either in parasitism or nematode metabolism. Although the available unigene set is not complete enough to analyze the whole life cycle of incompatible infection, it still serves as sequences and functions integrated resource for further analysis and identification of some pivotal parasitism genes and specific regulatory pathways during early CCN invasion, and there will benefit better in-depth understanding the molecular mechanisms of CCN parasitism. Moreover, it also provided candidates for improving the CCN resistance in crops by engineering techniques. Functional validation of individual candidates and their respective roles in CCN parasitism are needed to be further investigated.

## Supporting Information

S1 Fig Roots invaded by CCN. (PPTX)

S2 Fig Distribution of clusters by number of assembles Unigenes per cluster. (PPTX)

S3 Fig Classification of contigs/unigenes with significant similarity to nematode expressed sequence tags (ESTs) according to the nematode’s lifestyle. (PPTX)

S4 Fig Gene Ontology (GO) analysis of CCN genes using REVIGO. (PPTX)

S5 Fig Microscopic examination of lethal phenotypes of CCN under siRNA (25 μg/l) treatments. (PPTX)

S1 Table Downloaded nematode EST data including the species name. (DOCX)

S2 Table Summarizing putative homologues. (DOCX)

S3 Table Primer sequences used to validate putative effector and parasitism unigenes analyzing in our database. (DOCX)

S4 Table Nematode-like unigenes list in the transcriptome database. (XLSX)

S5 Table Expression level and annotation of CCN-like unigenes. (XLS)

S6 Table Identified proteins between our *H*. *avenae* (*Ha*) unigenes and the predicted proteins from the *M*. *incognita* (*Mi*) and *M*. *hapla*(*Mh*) genomes and (*H*. *glycines* and *H*.*avenea*) nematode unigenes. (DOCX)

S7 Table KEGG pathway enrichment. (XLS)

S8 Table Unigenes Cluster by STEM analysis. (XLSX)

S9 Table Selected protein using in PPI Network. (XLS)
