# Supplementary material for: RNA-Seq Based Identification of Candidate Parasitism Genes of Cereal Cyst Nematode (Heterodera avenae) during Incompatible Infection to Aegilops variabilis
Source: PLoS One. 2015 Oct 30;10(10):e0141095. doi: 10.1371/journal.pone.0141095 (PMC4627824; doi:10.1371/journal.pone.0141095)
Supplement: S1 Fig — (PPTX) [file pone.0141095.s001.pptx]

## Slide 1
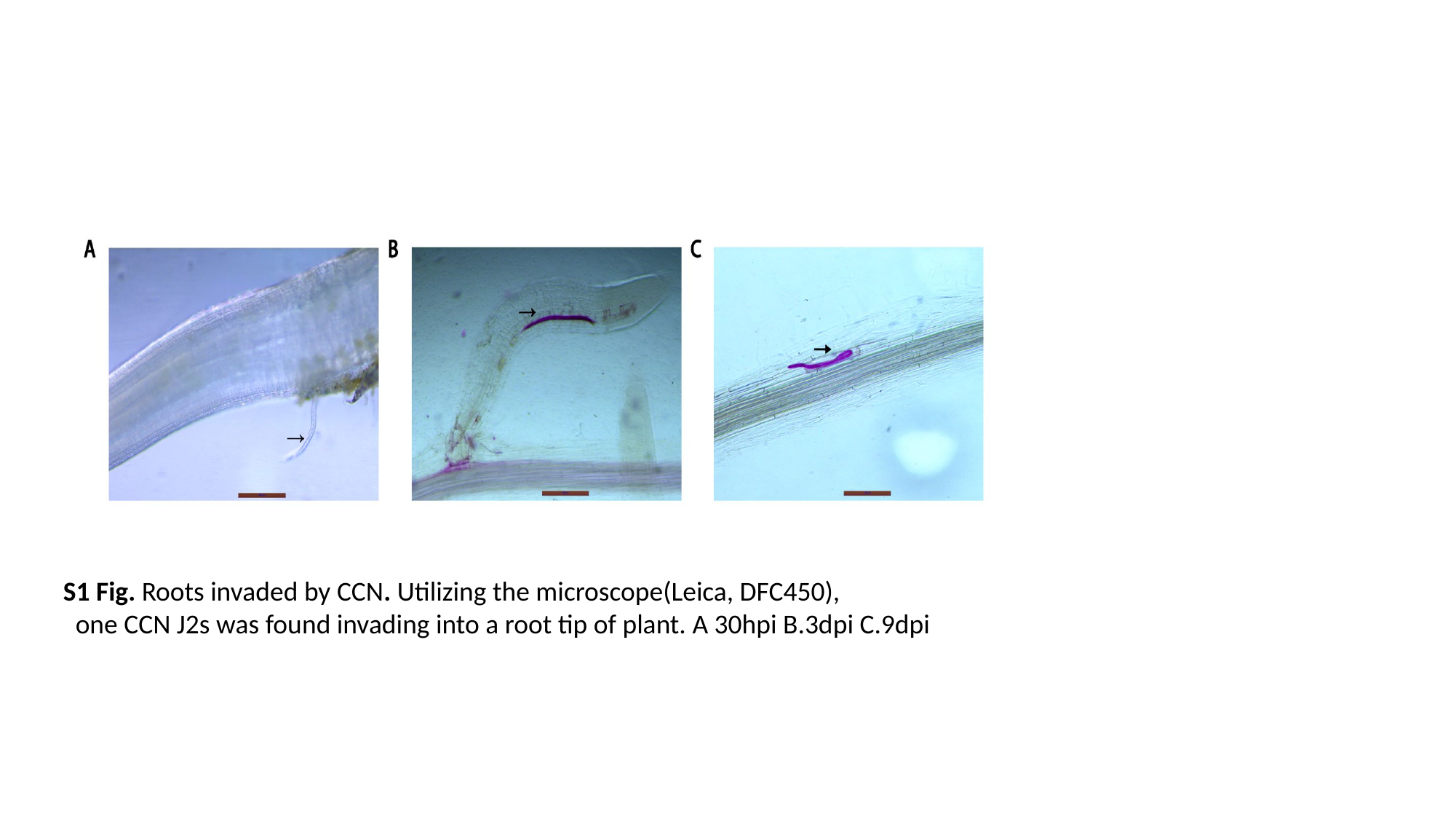

S1 Fig. Roots invaded by CCN. Utilizing the microscope(Leica, DFC450),
 one CCN J2s was found invading into a root tip of plant. A 30hpi B.3dpi C.9dpi
