# Supplementary material for: RNA-Seq Based Identification of Candidate Parasitism Genes of Cereal Cyst Nematode (Heterodera avenae) during Incompatible Infection to Aegilops variabilis
Source: PLoS One. 2015 Oct 30;10(10):e0141095. doi: 10.1371/journal.pone.0141095 (PMC4627824; doi:10.1371/journal.pone.0141095)
Supplement: S2 Fig — (PPTX) [file pone.0141095.s002.pptx]

## Slide 1
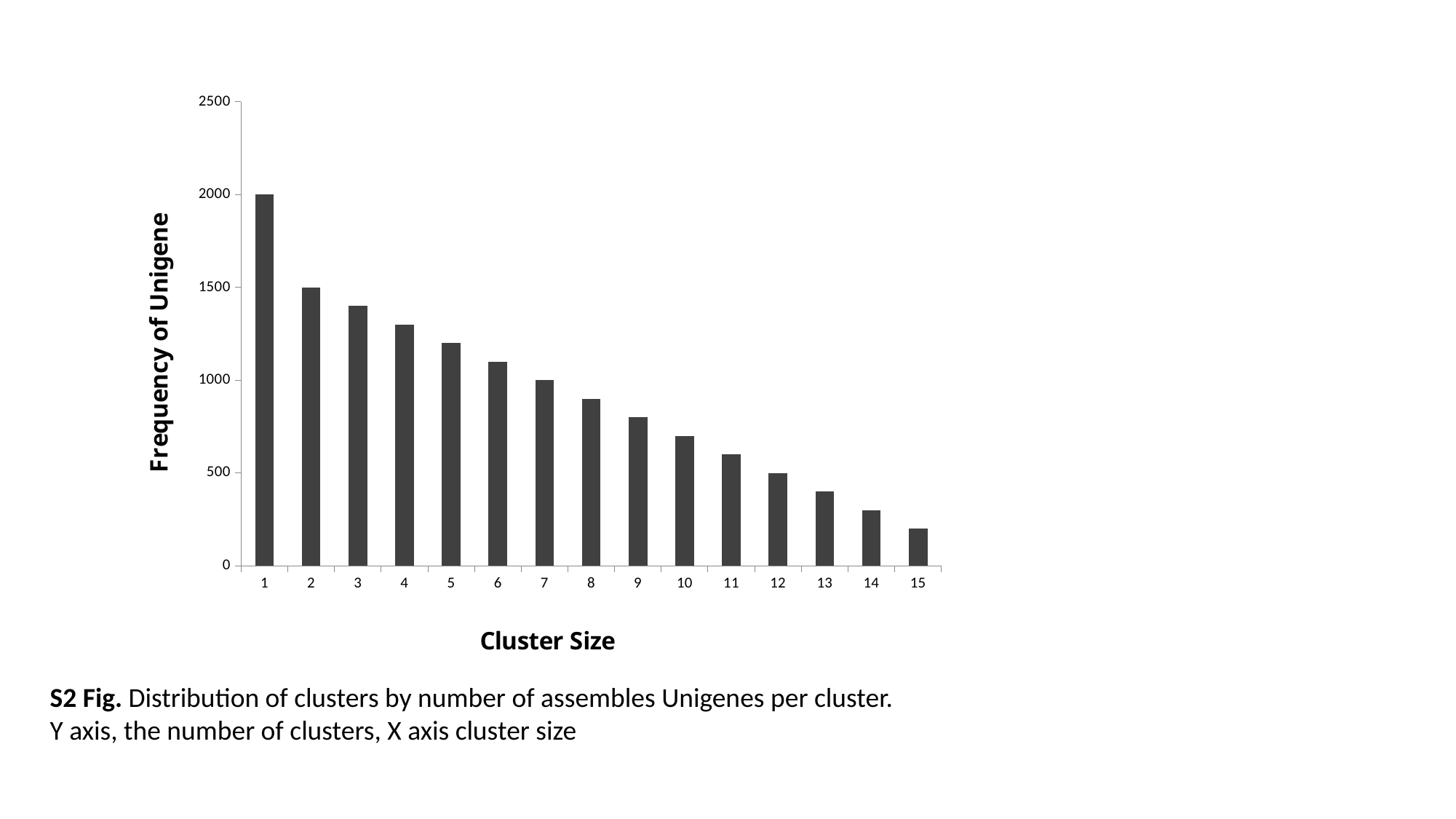

### Chart
| Category | |
|---|---|S2 Fig. Distribution of clusters by number of assembles Unigenes per cluster.
Y axis, the number of clusters, X axis cluster size
