# Supplementary material for: RNA-Seq Based Identification of Candidate Parasitism Genes of Cereal Cyst Nematode (Heterodera avenae) during Incompatible Infection to Aegilops variabilis
Source: PLoS One. 2015 Oct 30;10(10):e0141095. doi: 10.1371/journal.pone.0141095 (PMC4627824; doi:10.1371/journal.pone.0141095)
Supplement: S3 Fig — (PPTX) [file pone.0141095.s003.pptx]

## Slide 1
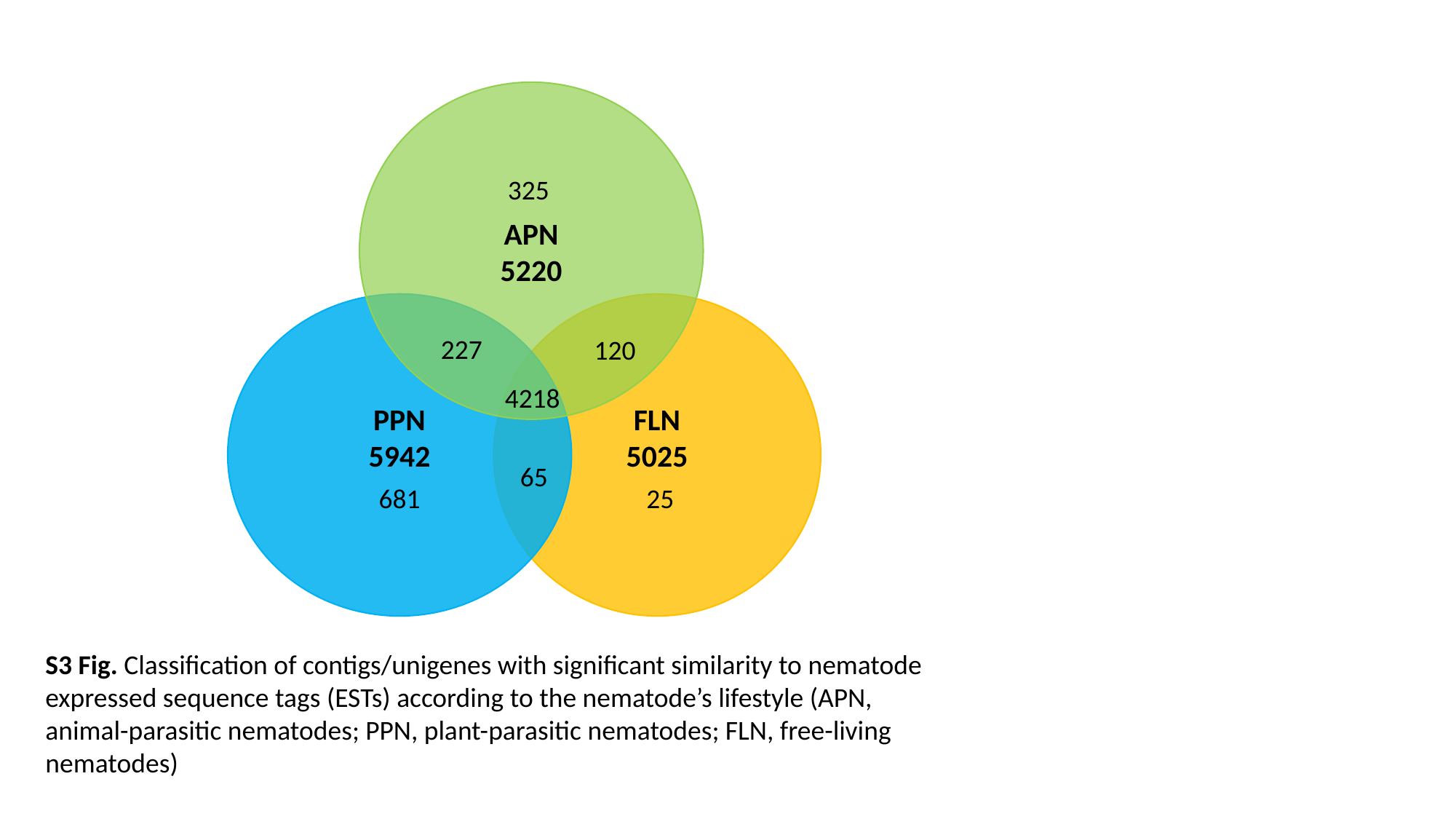

APN
5220
325
PPN
5942
FLN
5025
227
120
4218
65
681
25
S3 Fig. Classification of contigs/unigenes with significant similarity to nematode
expressed sequence tags (ESTs) according to the nematode’s lifestyle (APN,
animal-parasitic nematodes; PPN, plant-parasitic nematodes; FLN, free-living
nematodes)
