# Supplementary material for: RNA-Seq Based Identification of Candidate Parasitism Genes of Cereal Cyst Nematode (Heterodera avenae) during Incompatible Infection to Aegilops variabilis
Source: PLoS One. 2015 Oct 30;10(10):e0141095. doi: 10.1371/journal.pone.0141095 (PMC4627824; doi:10.1371/journal.pone.0141095)
Supplement: S4 Fig — (PPTX) [file pone.0141095.s004.pptx]

## Slide 1
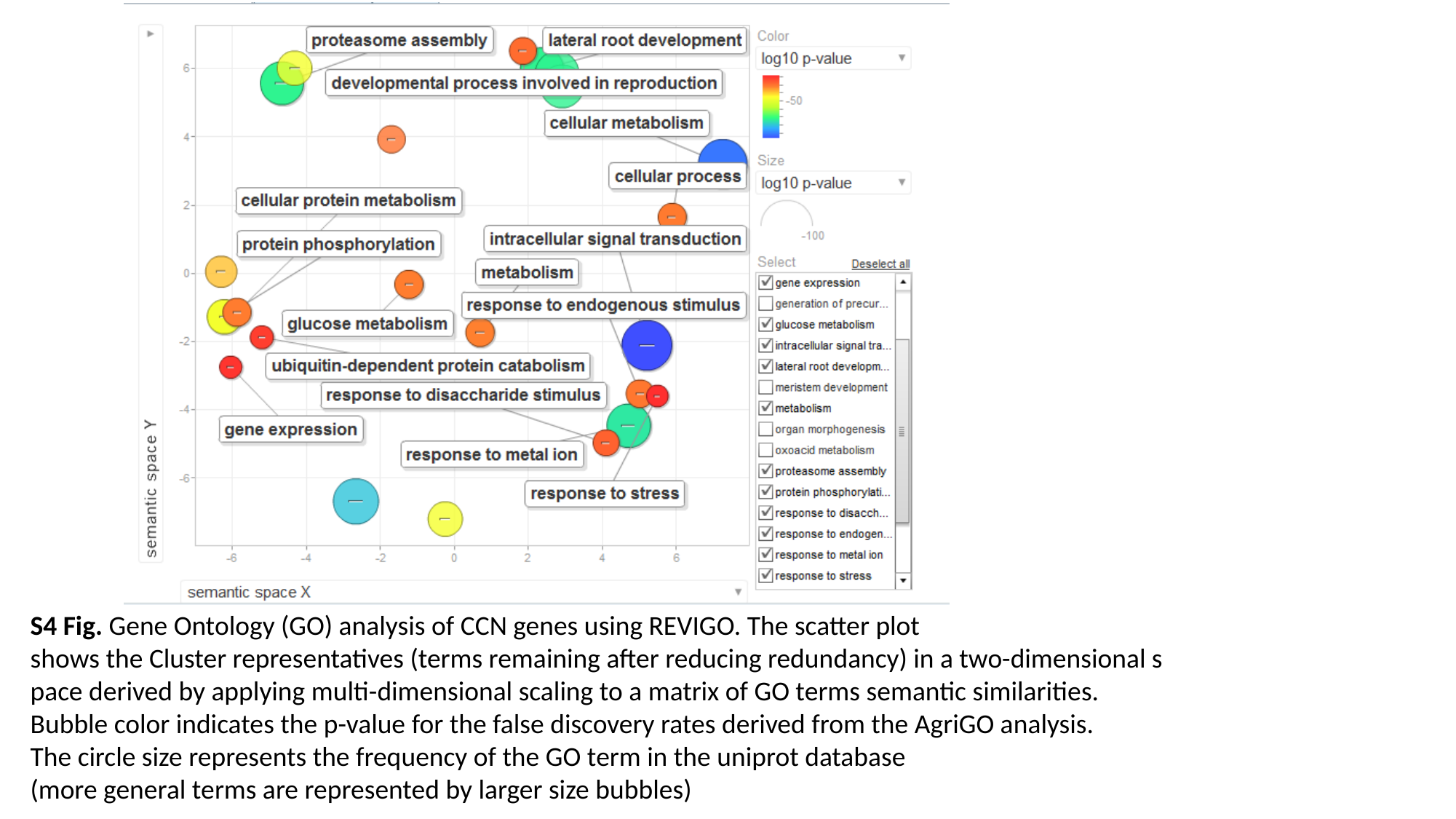

S4 Fig. Gene Ontology (GO) analysis of CCN genes using REVIGO. The scatter plot
shows the Cluster representatives (terms remaining after reducing redundancy) in a two-dimensional s
pace derived by applying multi-dimensional scaling to a matrix of GO terms semantic similarities.
Bubble color indicates the p-value for the false discovery rates derived from the AgriGO analysis.
The circle size represents the frequency of the GO term in the uniprot database
(more general terms are represented by larger size bubbles)
