# Supplementary material for: RNA-Seq Based Identification of Candidate Parasitism Genes of Cereal Cyst Nematode (Heterodera avenae) during Incompatible Infection to Aegilops variabilis
Source: PLoS One. 2015 Oct 30;10(10):e0141095. doi: 10.1371/journal.pone.0141095 (PMC4627824; doi:10.1371/journal.pone.0141095)
Supplement: S5 Fig — (PPTX) [file pone.0141095.s005.pptx]

## Slide 1
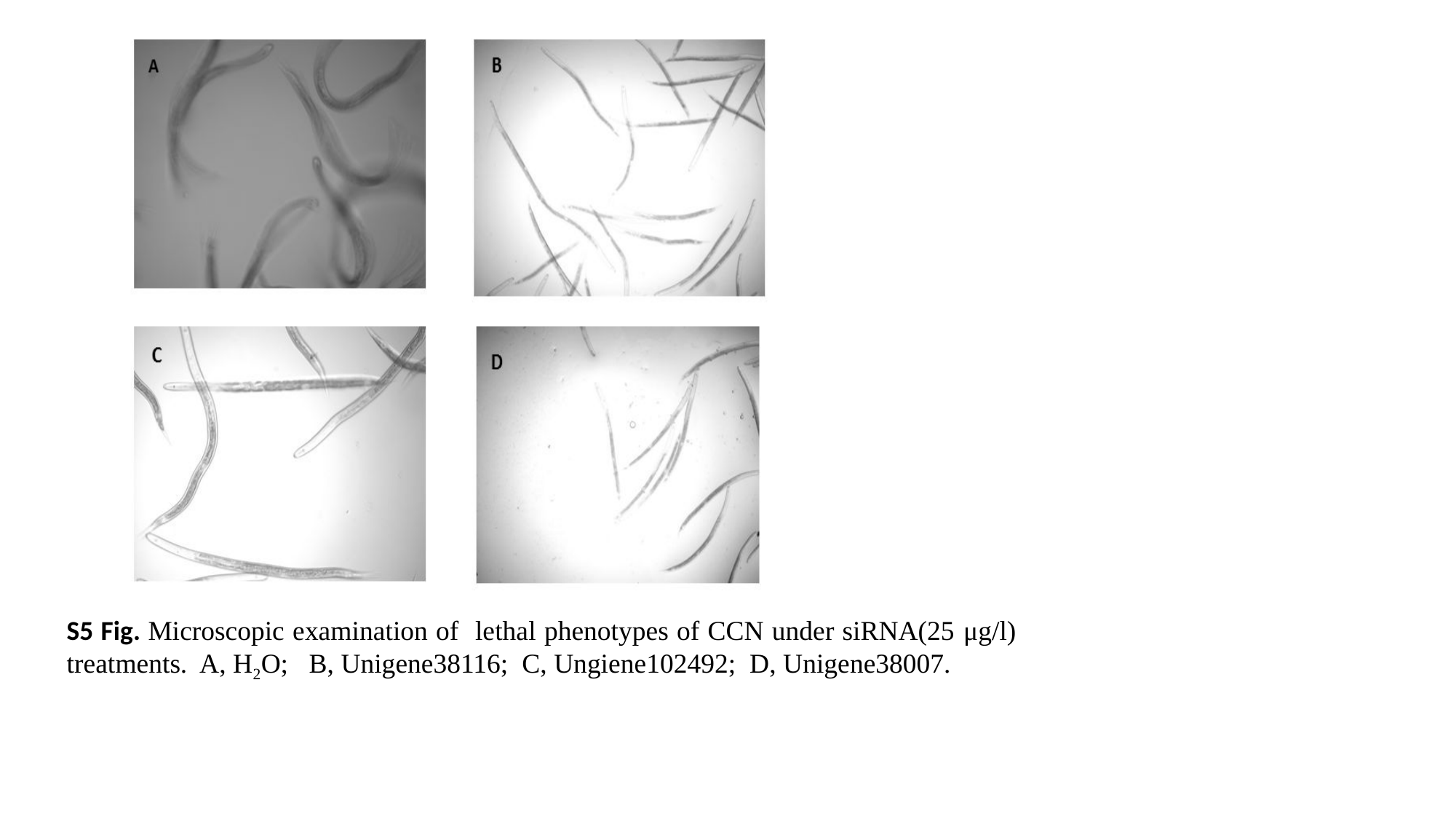

S5 Fig. Microscopic examination of lethal phenotypes of CCN under siRNA(25 μg/l) treatments. A, H2O; B, Unigene38116; C, Ungiene102492; D, Unigene38007.
