# Supplementary material for: RNA-Seq Based Identification of Candidate Parasitism Genes of Cereal Cyst Nematode (Heterodera avenae) during Incompatible Infection to Aegilops variabilis
Source: PLoS One. 2015 Oct 30;10(10):e0141095. doi: 10.1371/journal.pone.0141095 (PMC4627824; doi:10.1371/journal.pone.0141095)
Supplement: S1 Table — (DOCX) [file pone.0141095.s006.docx]

S1 Table Downloaded nematode EST data including the species name, the number of ESTs, the lifestyle of the nematode, the NCBI txid and the database.

| **nematode species** | **number of ESTs (02/2012)** | **lifestyle** | **NCBI txid** | **database** |
| --- | --- | --- | --- | --- |
| *Ancylostoma braziliense* | 20 | APN | 369059 | NCBI dbEST |
| *Ancylostoma caninum* | 80905 | APN | 29170 | NCBI dbEST |
| *Ancylostoma ceylanicum* | 10651 | APN | 53326 | NCBI dbEST |
| *Angiostrongylus cantonensis* | 2622 | APN | 6313 | NCBI dbEST |
| *Anisakis simplex* | 475 | APN | 6269 | NCBI dbEST |
| *Ascaris lumbricoides* | 1822 | APN | 6252 | NCBI dbEST |
| *Ascaris suum* | 56616 | APN | 6253 | NCBI dbEST |
| *Brugia malayi* | 26215 | APN | 6279 | NCBI dbEST |
| *Brugia pahangi* | 28 | APN | 6280 | NCBI dbEST |
| *Dictyocaulus viviparus* | 6264 | APN | 29172 | NCBI dbEST |
| *Dirofilaria immitis* | 4005 | APN | 6287 | NCBI dbEST |
| *Haemonchus contortus* | 21975 | APN | 6289 | NCBI dbEST |
| *Heterorhabditis bacteriophora* | 53614 | APN | 37862 | NCBI dbEST |
| *Litomosoides sigmodontis* | 2699 | APN | 42156 | NCBI dbEST |
| *Loa loa* | 4173 | APN | 7209 | NCBI dbEST |
| *Mecistocirrus digitatus* | 1 | APN | 237660 | NCBI dbEST |
| *Necator americanus* | 6694 | APN | 51031 | NCBI dbEST |
| *Nippostrongylus brasiliensis* | 14686 | APN | 27835 | NCBI dbEST |
| *Oesophagostomum dentatum* | 299 | APN | 61180 | NCBI dbEST |
| *Onchocerca flexuosa* | 2124 | APN | 387005 | NCBI dbEST |
| *Onchocerca ochengi* | 60 | APN | 42157 | NCBI dbEST |
| *Onchocerca volvulus* | 14974 | APN | 6282 | NCBI dbEST |
| *Ostertagia ostertagi* | 7006 | APN | 6317 | NCBI dbEST |
| *Parastrongyloides trichosuri* | 7963 | APN | 131310 | NCBI dbEST |
| *Parelaphostrongylus tenuis* | 99 | APN | 148309 | NCBI dbEST |
| *Steinernema carpocapsae* | 2218 | APN | 34508 | NCBI dbEST |
| *Steinernema feltiae* | 83 | APN | 52066 | NCBI dbEST |
| *Strongyloides ratti* | 27366 | APN | 34506 | NCBI dbEST |
| *Strongyloides stercoralis* | 11392 | APN | 6248 | NCBI dbEST |
| *Teladorsagia circumcincta* | 6061 | APN | 45464 | NCBI dbEST |
| *Toxascaris leonina* | 439 | APN | 59264 | NCBI dbEST |
| *Toxocara canis* | 5069 | APN | 6265 | NCBI dbEST |
| *Trichinella pseudospiralis* | 17330 | APN | 6337 | NCBI dbEST |
| *Trichinella spiralis* | 25268 | APN | 6334 | NCBI dbEST |
| *Trichostrongylus vitrinus* | 368 | APN | 40352 | NCBI dbEST |
| *Trichuris muris* | 7102 | APN | 70415 | NCBI dbEST |
| *Trichuris vulpis* | 3063 | APN | 219738 | NCBI dbEST |
| *Wuchereria bancrofti* | 4847 | APN | 6293 | NCBI dbEST |
| *Caenorhabditis brenneri* | 29997 | FLN | 135651 | NCBI dbEST |
| *Caenorhabditis briggsae* | 2424 | FLN | 6238 | NCBI dbEST |
| *Caenorhabditis elegans* | 396687 | FLN | 6239 | NCBI dbEST |
| *Caenorhabditis japonica* | 33050 | FLN | 281687 | NCBI dbEST |
| *Caenorhabditis remanei* | 20292 | FLN | 31234 | NCBI dbEST |
| *Panagrolaimus davidi* | 1 | FLN | 227884 | NCBI dbEST |
| *Panagrolaimus superbus* | 7609 | FLN | 310955 | NCBI dbEST |
| *Plectus murrayi* | 2591 | FLN | 538668 | NCBI dbEST |
| *Pristionchus pacificus* | 37470 | FLN | 54126 | NCBI dbEST |
| *Zeldia punctata* | 391 | FLN | 49351 | NCBI dbEST |
| *Aphelenchus avenae* | 5119 | PPN | 70226 | NCBI dbEST |
| *Bursaphelenchus mucronatus* | 3193 | PPN | 6325 | NCBI dbEST |
| *Bursaphelenchus xylophilus* | 14059 | PPN | 6326 | NCBI dbEST |
| *Ditylenchus africanus* | 4847 | PPN | 490491 | NCBI dbEST |
| *Globodera mexicana* | 17 | PPN | 182293 | NCBI dbEST |
| *Globodera pallida* | 9020 | PPN | 36090 | NCBI dbEST |
| *Globodera rostochiensis* | 11851 | PPN | 31243 | NCBI dbEST |
| *Heterodera avenae* | 27,765 | PPN |  | HATdb |
| *Heterodera glycines* | 24444 | PPN | 51029 | NCBI dbEST |
| *Heterodera schachtii* | 2812 | PPN | 97005 | NCBI dbEST |
| *Meloidogyne arenaria* | 5042 | PPN | 6304 | NCBI dbEST |
| *Meloidogyne chitwoodi* | 12218 | PPN | 59747 | NCBI dbEST |
| *Meloidogyne hapla* | 24452 | PPN | 6305 | NCBI dbEST |
| *Meloidogyne incognita* | 20334 | PPN | 6306 | NCBI dbEST |
| *Meloidogyne javanica* | 7587 | PPN | 6303 | NCBI dbEST |
| *Meloidogyne paranaensis* | 3710 | PPN | 189293 | NCBI dbEST |
| *Pratylenchus penetrans* | 1916 | PPN | 45929 | NCBI dbEST |
| *Pratylenchus vulnus* | 5812 | PPN | 45931 | NCBI dbEST |
| *Radopholus similis* | 7382 | PPN | 46012 | NCBI dbEST |
