# Supplementary material for: RNA-Seq Based Identification of Candidate Parasitism Genes of Cereal Cyst Nematode (Heterodera avenae) during Incompatible Infection to Aegilops variabilis
Source: PLoS One. 2015 Oct 30;10(10):e0141095. doi: 10.1371/journal.pone.0141095 (PMC4627824; doi:10.1371/journal.pone.0141095)
Supplement: S2 Table — (DOCX) [file pone.0141095.s007.docx]

S2 Table Table summarizing putative homologues to all currently known putative effectors including their (speculative) function, accession number, number of *H. avenae* unigenes with hits, number of reads corresponding to these contigs, bit score in blast, the species in which the effector has been found (Aa: *Aphelenchus avenae*, Af: *Aphelenchoides fragariae*, Bm: *Bursaphelenchus mucronatus*, Bx: *Bursaphelenchus xylophilus*, Da: *Ditylenchus africanus*, Gm: *Globodera mexicana*, Gp: *Globodera pallida*, Gr: *Globodera rostochiensis*, Gt: *Globodera tabacum*, Hg: *Heterodera glycines*, Hs: *Heterodera schachtii*, Ma: *Meloidogyne arenaria*, Mc: *Meloidogyne chitwoodi*, Mh: *Meloidogyne hapla*, Mi: *Meloidogyne incognita*, Mj: *Meloidogyne javanica*, Pc: *Pratylenchus coffeae*, Pp: *Pratylenchus penetrans*, Rr: *Rotylenchulus reniformis*, Rs: *Radopholus similis*, Xi: *Xiphinema index*) and corresponding references.

| **Effector** | **(Speculative) function** | **Accession nr** | **# *H. graminicola* contigs with hit** | **bit score** | **Species** | **References** |
| --- | --- | --- | --- | --- | --- | --- |
| GHF5 beta-1,4-endoglucanase | plant cell wall degradation | Q9UA57 | No hits |  | Aa, Af, Da, Gp, Gr, Gt, Hg, Hs, Mc, Mh, Mi, Pc, Pp, Rr, Rs | Bakhetia et al., 2007; Bellafiore et al., 2008; Bera-Maillet et al., 2000; De Meutter et al., 2001; Fu et al., 2012; Gao et al., 2002a; Gao et al., 2003; Goellner et al., 2000; Haegeman et al., 2008; Haegeman et al., 2010; Haegeman et al., 2011; Jones et al., 2009; Karim et al., 2009; Ledger et al., 2006; Rehman et al., 2009a; Rosso et al., 1999; Roze et al., 2008; Smant et al., 1998; Uehara et al., 2001; Vanholme et al., 2006; Wang et al., 1999; Wubben et al., 2010; Yan et al., 1998; Yan et al., 2001 |
| GHF45 beta-1,4-endoglucanase | plant cell wall degradation | AFG30029 | no hits |  | Bx | Kikuchi et al., 2004 |
| beta-1,3-endoglucanase | plant cell wall degradation | BAE48357 | no hits |  | Aa, Bm, Bx, Pc | Haegeman et al., 2011; Karim et al., 2009; Kikuchi et al., 2005 |
| cellulose binding protein | plant cell wall degradation | A2VBB1 | No hits |  | Gp, Hg, Hs, Mh, Mi, Mj, Pc | Adam et al., 2008; Ding et al., 1998; Gao et al., 2003; Gao et al., 2004; Hewezi et al., 2008; Huang et al., 2003; Ithal et al., 2007; Jones et al., 2009; Opperman et al., 2008; Vanholme et al., 2006 |
| pectate lyase | plant cell wall degradation | AAQ09004 | No hits |  | Aa, Bx, Hg, Hs, Mc, Mh, Mi, Mj, Pc | Bakhetia et al., 2007; Bellafiore et al., 2008; de Boer et al., 2002a; Doyle and Lambert, 2002; Gao et al., 2003; Haegeman et al., 2011; Huang et al., 2005a; Karim et al., 2009; Kikuchi et al., 2006; Popeijus et al., 2000a; Roze et al., 2008; Vanholme et al., 2006; Vanholme et al., 2007; Vieira et al., 2011 |
| arabinogalactan endo-1,4-beta-galactosidase | plant cell wall degradation | ACY02855 | no hits |  | Hs, Pc | Haegeman et al., 2011; Vanholme et al., 2006; Vanholme et al., 2009a |
| polygalacturonase | plant cell wall degradation | AAM28240 | no hits |  | Mh, Mi, Pc | Bellafiore et al., 2008; Haegeman et al., 2011; Jaubert et al., 2002a; Opperman et al., 2008 |
| xylanase | plant cell wall degradation | AAF37276 | 1 | 107 | Mh, Mi, Pc, Rs, | Haegeman et al., 2009b; Haegeman et al., 2011; Jacob et al., 2008; Mitreva-Dautova et al., 2006; Opperman et al., 2008 |
| expansin | plant cell wall extension | ADX36366 | 2 | 85.9 | Bx, Bm, Da, Gp, Gr, Mh, Mi, Mj, Pc | Abad et al., 2008; Bellafiore et al., 2008; Haegeman et al., 2010; Haegeman et al., 2011; Jones et al., 2009; Kikuchi et al., 2009; Kudla et al., 2005; Opperman et al., 2008; Qin et al., 2004 |
| peroxiredoxin | detoxification of ROS | CAB48391 | 7 | 262 | Da, Gr, Hg, Mh, Mi, Pc, Xi | Dautova et al., 2001; Dubreuil et al., 2011; Haegeman et al., 2009a; Haegeman et al., 2011; Opperman et al., 2008; Robertson et al., 2000 |
| glutathione peroxidase | detoxification of ROS | CAD38523 | 7 | 244 | Gr, Hs, Mh, Mi, Pc, Rs | Bellafiore et al., 2008; Dautova et al., 2001; Haegeman et al., 2011; Jacob et al., 2008; Jones et al., 2004; Opperman et al., 2008; Popeijus et al., 2000b; Vanholme et al., 2006 |
| glutathione-S-transferase | detoxification of ROS | ABN64198 | 15 | 213 | Da, Mi, Pc, Rs | Bellafiore et al., 2008; Dubreuil et al., 2007; Haegeman et al., 2009a; Haegeman et al., 2011; Jacob et al., 2008 |
| fatty acid and retinol binding protein (FAR) | binding of host fatty acids reducing defense response | CAA70477 | 1 | 174 | Da, Gp, Gr, Hs, Mc, Mh, Mi, Rs, Pc | Bellafiore et al., 2008; Haegeman et al., 2009a; Haegeman et al., 2011; Jacob et al., 2008; Opperman et al., 2008; Popeijus et al., 2000b; Prior et al., 2001; Roze et al., 2008; Vanholme et al., 2006 |
| chorismate mutase | plant defense suppression by reduction of SA levels | AAD42163 | no hits |  | Gp, Hg, Hs, Ma, Mh, Mi, Mj, Pp | Bakhetia et al., 2007; Bekal et al., 2003; Doyle and Lambert, 2003; Gao et al., 2003; Haegeman et al., 2011; Huang et al., 2005b; Jones et al., 2009; Long et al., 2006; Opperman et al., 2008; Popeijus et al., 2000b; Vanholme et al., 2009b |
| SPRYSECs | plant defense suppression, potential avirulence genes | CAM33004 | no hits |  | Gm, Gp, Gr | Blanchard et al., 2005; Jones et al., 2009; Qin et al., 2000; Rehman et al., 2009b; Sacco et al., 2009 |
| 30C02 | plant defense suppression | AAP30836 | no hits |  | Hg, Hs | Gao et al. 2003; Hamamouch et al., 2012 |
| CLE peptide | mimicking plant CLEs: involved in syncytium formation | AAO33474 | no hits |  | Gr, Hg, Hs | Guo et al., 2010; Guo et al., 2011; Lu and Wang, 2006; Lu et al., 2009; Olsen and Skriver, 2003; Replogle et al., 2009; Replogle et al., 2011; Wang et al., 2010a; Wang et al., 2011; Wang et al., 2006; Wang et al., 2010b; Wang et al., 2005 |
| 16D10 CLE related protein | transcriptional regulation to promote giant cell induction | Q06JG6 | no hits |  | Ma, Mh, Mi, Mj | Huang et al., 2006b; Huang et al., 2006a |
| 19C07 | modification of auxin influx in syncytia | AAO85458 | no hits |  | Hg, Hs | Lee et al., 2011 |
| annexin | mimicking plant annexin: protection of cells against stress | AAN32888 | 3 | 124 | Gp, Hg, Hs, Mh | Jones et al., 2009; Opperman et al., 2008; Patel et al., 2010 |
| calreticulin | calcium signalling | AAL40720 | 6 | 329 | Hg, Mh, Mi | Ithal et al., 2007; Jaubert et al., 2002b; Jaubert et al., 2005; Opperman et al., 2008 |
| 10A06 = RING-H2 | indirect induction of antioxidant genes in syncytium | ACU12489 | no hits |  | Hg, Hs | Gao et al. 2003; Hewezi et al., 2010; Sindhu et al., 2009 |
| SKP-1 | involved in signal transduction | AAP30763 | no hits with signal peptide |  | Hg, Mi | Bellafiore et al., 2008; Gao et al., 2003 |
| ubiquitin extension protein | selective protein degradation | AAO33478 | no hits with signal peptide |  | Gp, Hg, Hs | Gao et al., 2003; Jones et al., 2009; Tytgat et al., 2004 |
| chitinase | egg hatching | AAN14978 | no hits |  | Aa, Bx, Hg, Hs, Mh, Mi | Dautova et al., 2001; Gao et al., 2002b; Karim et al., 2009; Kikuchi et al., 2007; Opperman et al., 2008; Vanholme et al., 2006 |
| transthyretin-like proteins | growth regulation | CAM84510 | 16 | 119 | Da, Gp, Hg, Mi, Pc, Rs, Xi | Bellafiore et al., 2008; Furlanetto et al., 2005; Gao et al., 2003; Haegeman et al., 2009a; Haegeman et al., 2011; Jacob et al., 2007; Jones et al., 2009; McCarter et al., 2003 |
| MAP-1 | recognition between plant and nematode | CAC27774 | 1 | 28 | Mc, Mh | Roze et al., 2008; Semblat et al., 2001; Vieira et al., 2011 |
| venom allergen proteins | recognition between plant and nematode | CAD60978 | no hits |  | Bx, Da, Gp, Gr, Hg, Hs, Ma, Mc, Mh, Mi, Mj, Pc | Ding et al., 2000; Gao et al., 2001; Haegeman et al., 2009a; Haegeman et al., 2011; Jones et al., 2009; Kang et al., 2010; Lozano-Torres et al., 2012; Opperman et al., 2008; Roze et al., 2008; Vanholme et al., 2006; Wang et al., 2007 |
| Mj-NULG1a | unknown | AFB73917 | no hits |  | Mj | Lin et al., 2012 |
| 7E12 | unknown | AAQ10021 | no hits |  | Mi | de Lima e Souza et al., 2011; Huang et al., 2003 |
| galectin | unknown | AAB61596 | no hits |  | Mi, Rs | Dubreuil et al., 2007; Jacob et al., 2008 |
| C-type lectin | unknown | AAM18623 | 3 | 57.4 | Hg, Mc | de Boer et al., 2002b; Roze et al., 2008; Urwin et al., 2002 |
| 14-3-3 | unknown | AAL40719 | 8 | 306 | Mh, Mi | Bellafiore et al., 2008; Jaubert et al., 2004; Jaubert et al., 2002b; Opperman et al., 2008 |
| SXP-RAL2 | unknown | CAB75701 | no hits |  | Gr, Mh, Mi, Pc | Haegeman et al., 2011; Jones et al., 2000; Opperman et al., 2008; Tytgat et al., 2005 |
| 2E07 | unknown | AAQ10015 | no hits |  | Mi | Huang et al. 2003 |
| 2G02 | unknown | AAQ10016 | no hits |  | Mi | Huang et al. 2003 |
| 2G10 | unknown | AAN15807 | no hits |  | Mi | Huang et al. 2003 |
| 4D01 | unknown | AAQ10017 | no hits |  | Mi | Huang et al. 2003 |
| 4D03 | unknown | AAN15808 | no hits |  | Mi | Huang et al. 2003 |
| 5G05 | unknown | AAN15806 | no hits |  | Mi | Huang et al. 2003 |
| 6F06 | unknown | AAQ10018 | no hits |  | Mi | Huang et al. 2003 |
| 6G07 | unknown | AAQ10019 | no hits |  | Mi | Huang et al. 2003 |
| 7A01 | unknown | AAQ10020 | no hits |  | Mi | Huang et al. 2003 |
| 7H08 | unknown | AF531168 | no hits |  | Mi | Huang et al. 2003 |
| 8D05 | unknown | AF531169 | no hits |  | Mi | Huang et al. 2003 |
| 8H11 | unknown | AF531170 | no hits |  | Mi | Huang et al. 2003 |
| 9H10 | unknown | AF531167 | no hits |  | Mi | Huang et al. 2003 |
| 10A08 | unknown | AY142117 | no hits |  | Mi | Huang et al. 2003 |
| 10G02 | unknown | AY135365 | no hits |  | Mi | Huang et al. 2003 |
| 11A01 | unknown | AY134431 | no hits |  | Mi | Huang et al. 2003 |
| 12H03 | unknown | AY134432 | no hits |  | Mi | Huang et al. 2003 |
| 13A12 | unknown | AY134433 | no hits |  | Mi | Huang et al. 2003 |
| 14E06 | unknown | AY134434 | no hits |  | Mi | Huang et al. 2003 |
| 16E05 | unknown | AY134436 | no hits |  | Mi | Huang et al. 2003 |
| 17H02 | unknown | AY134437 | no hits |  | Mi | Huang et al. 2003 |
| 19F07 | unknown | AY142116 | no hits |  | Mi | Huang et al. 2003 |
| 21E02 | unknown | AY134438 | no hits |  | Mi | Huang et al. 2003 |
| 25B10 | unknown | AY142118 | no hits |  | Mi | Huang et al. 2003 |
| 28B04 | unknown | AY142119 | no hits |  | Mi | Huang et al. 2003 |
| 30G11 | unknown | AY134440 | no hits |  | Mi | Huang et al. 2003 |
| 30H07 | unknown | AY134439 | no hits |  | Mi | Huang et al. 2003 |
| 31H06 | unknown | AY134441 | no hits |  | Mi | Huang et al. 2003 |
| 34D01 | unknown | AY134442 | no hits |  | Mi | Huang et al. 2003 |
| 34F06 | unknown | AY134443 | no hits |  | Mi | Huang et al. 2003 |
| 35A02 | unknown | AY134444 | no hits |  | Mi | Huang et al. 2003 |
| 35E04 | unknown | AY142121 | no hits |  | Mi | Huang et al. 2003 |
| 35F03 | unknown | AY142120 | no hits |  | Mi | Huang et al. 2003 |
