# Supplementary material for: RNA-Seq Based Identification of Candidate Parasitism Genes of Cereal Cyst Nematode (Heterodera avenae) during Incompatible Infection to Aegilops variabilis
Source: PLoS One. 2015 Oct 30;10(10):e0141095. doi: 10.1371/journal.pone.0141095 (PMC4627824; doi:10.1371/journal.pone.0141095)
Supplement: S3 Table — (DOCX) [file pone.0141095.s008.docx]

S3 Table Primer sequences used to validate putative effectors and parasitism unigenes analyzed in our database

| **Primer name** | **Primer sequence (5’ to 3’)** | **Length(bp)** |
| --- | --- | --- |
| Unigene38560-F | TGTCTGATTGTCCTTCCTCCTTGGCCTTTT | 130 |
| Unigene38560-R | CATTCTTGTTGCTGGACGACGAAGTTGC |  |
| Unigene103494-F | ATAGCGTTTCATGTCCGATGACCATTTC | 166 |
| Unigene103494-R | TTGGCAGTCTGTTCCATCAGTTATTTCG |  |
| Unigene69027-F | CCTTGCGTGCTTCCTTCGGTTTGGGTGG | 190 |
| Unigene69027-R | TCGATTGGCATCTATGGGATGGACTTCT |  |
| Unigene66286-F | GTTCATTCGTAAACCCATGAGTTCTGCG | 100 |
| Unigene66286-R | TGAGCGAGAAATCGTCCATTGGTTCAAG |  |
| Unigene87881-F | CATGGAATCTTTCGGATTCACTGCTGAT | 165 |
| Unigene87881-R | TTAAGTCCTTTTCTGACTCTTGTTTTGT |  |
| Unigene58375-F | AGAGCAATAACCCCTGTAACGCTGATAA | 132 |
| Unigene58375-R | GAAAACAAAGGCAGTGACAAGGCCGAAA |  |
| Unigene49109-F | AAGACGGATCAGAACCTGACCATTTACG | 154 |
| Unigene49109-R | ACGGGTTGAAGTCGATGTACTGCTGAGTGT |  |
| Unigene70977-F | GGCAACCTCAGCCAAATAGCGATAGTAGTCAC | 206 |
| Unigene70977-R | TTGAGCAGAAGACAGAAGGCTCGGAAAA |  |
| Unigene107298-F | CCGTGCTGATTGTGAGGATAGAAAGTGA | 100 |
| Unigene107298-R | GAGGACAAGAAGAAGGTGGAGGAAGAGG |  |
