# Supplementary material for: RNA-Seq Based Identification of Candidate Parasitism Genes of Cereal Cyst Nematode (Heterodera avenae) during Incompatible Infection to Aegilops variabilis
Source: PLoS One. 2015 Oct 30;10(10):e0141095. doi: 10.1371/journal.pone.0141095 (PMC4627824; doi:10.1371/journal.pone.0141095)
Supplement: S6 Table — (DOCX) [file pone.0141095.s011.docx]

**Supplement Table 6** This table showed identified proteins between our *H. avenae* (Ha) unigenes and the predicted proteins from the *M. incognita*(Mi) and *M. hapla*(Mh) genomes and (*H. glycines* and *H.avenae* ) nematode unigenes. The numbers in red represent the common sequences.

| **Species** | ***Unigene/Contig*** | **Common Sequence** |
| --- | --- | --- |
| *M. incognita* | 13059 | 235 |
| *M. hapla* | 7207 | 189 |
| *H. glycines* | 6860 | 312 |
| *H. avenae* | 27765 | 525 |
